# The Role of Biological Rhythms in New Drug Formulations to Cross the Brain Barriers

**DOI:** 10.3390/ijms241612541

**Published:** 2023-08-08

**Authors:** Rafael Mineiro, Tânia Albuquerque, Ana Raquel Neves, Cecília R. A. Santos, Diana Costa, Telma Quintela

**Affiliations:** 1 CICS-UBI—Health Sciences Research Centre, Universidade da Beira Interior, Avenida Infante D. Henrique, 6200-506 Covilhã, Portugal; 2 UDI-IPG—Unidade de Investigação para o Desenvolvimento do Interior, Instituto Politécnico da Guarda, 6300-559 Guarda, Portugal

**Keywords:** blood–brain barrier, blood–cerebrospinal fluid barrier, circadian rhythms, nanoformulations, nanoparticles, liposomes, nanotubes, exosomes

## Abstract

For brain protection, the blood–brain barrier and blood–cerebrospinal fluid barrier limit the traffic of molecules between blood and brain tissue and between blood and cerebrospinal fluid, respectively. Besides their protective function, brain barriers also limit the passage of therapeutic drugs to the brain, which constitutes a great challenge for the development of therapeutic strategies for brain disorders. This problem has led to the emergence of novel strategies to treat neurological disorders, like the development of nanoformulations to deliver therapeutic agents to the brain. Recently, functional molecular clocks have been identified in the blood–brain barrier and in the blood–cerebrospinal fluid barrier. In fact, circadian rhythms in physiological functions related to drug disposition were also described in brain barriers. This opens the possibility for chronobiological approaches that aim to use time to improve drug efficacy and safety. The conjugation of nanoformulations with chronobiology for neurological disorders is still unexplored. Facing this, here, we reviewed the circadian rhythms in brain barriers, the nanoformulations studied to deliver drugs to the brain, and the nanoformulations with the potential to be conjugated with a chronobiological approach to therapeutic strategies for the brain.

## 1. Introduction

Neurological disorders represent the second leading cause of death and the principal motive of disability-adjusted life-years. Disability-adjusted life-years correspond to the sum of the years lived with disability and the years of life lost [1]. Part of this problem is due to the difficulty of developing therapeutic agents to treat brain disorders [2]. This difficulty arises due to the presence of brain barriers that limit the traffic of molecules into the brain tissue. Brain barriers are specialized structures composed of endothelial or epithelial cells sealed by tight junctions. The epithelial and endothelial cells of the brain barriers express membrane transporters, membrane receptors, and detoxification enzymes, which, together with tight junctions, control the passage of substances into the brain. The blood–brain barrier (BBB) is the most important brain barrier and is composed of brain capillary endothelial cells. The BBB is responsible for limiting the traffic of molecules between blood and brain tissue [3]. In addition to the BBB, the blood–cerebrospinal fluid barrier (BCSFB) is composed of choroid plexus epithelial cells and limits the traffic of substances between the blood and the cerebrospinal fluid (CSF) contained in the brain ventricles [4].

Circadian rhythms are rhythmic daily oscillations in biological functions developed by living organisms to adapt to environmental changes [5,6]. These daily rhythms are driven by molecular clocks where specific genes (the clock genes) undergo transcriptional-translational feedback loops (TTFLs). The core loop involves the formation of a heterodimeric complex by the circadian locomotor output cycles kaput (CLOCK) and brain and muscle ARNT-like 1 (BMAL1) proteins, which regulate many clock-controlled genes (CCGs). Among those CCGs, the CLOCK-BMAL1 complex positively regulates the expression of the repressor regulators’ cryptochrome (*Cry*) and period (*Per*). The respective proteins of *Per* and *Cry* genes form a complex that interacts with the complex formed by CLOCK and BMAL1, leading to the repression of their own transcription [7]. The circadian system has a hierarchical organization where the suprachiasmatic nucleus of the hypothalamus (SCN) functions as the master clock. The SCN clock is responsible for receiving light cues from the retina and for the synchronization of the peripheral clocks across the body [8]. Functional circadian clocks were recently discovered and characterized in the BBB and in the BCSFB [9,10,11]. Circadian rhythms involved in some functions related to drug disposition in these two barriers were also reported. For example, in the BBB, ABCB1, also known as p-glycoprotein, showed a circadian function, and the transport of methotrexate across the BCSFB showed a daily oscillation [11,12]. This opens a pathway for the future development and improvement of chronobiological therapeutic strategies for neurological disorders which incorporate the best timing for drug administration in order to improve the efficacy and safety of a therapeutic agent.

To overcome the difficulty of developing treatments for brain disorders, the development of nanoformulations has emerged as a novel strategy to effectively deliver a therapeutic agent to the brain. For example, these nanoformulations can be nanoparticles, liposomes, or nanotubes. Nanoformulations like nanoparticles aim to improve bioavailability, increase the half-life, and target the therapeutic agent to a specific site [13]. Targeting a specific site normally involves the functionalization of the nanocarriers with a specific ligand for a membrane protein [14]. Furthermore, nanocarriers could also incorporate inhibitors of the efflux pumps [15]. According to this, for neurological disorders, nanoformulations can make use of the transport mechanisms across the brain barriers and evade the high efflux activity in these barriers.

Due to the challenges of developing pharmacological therapies for neurological disorders, novel therapeutic strategies have emerged and are still emerging. The development of nanoformulations and chronobiological approaches constitute a novelty in this field, and the connection between the two strategies is still unexplored. For example, nanoformulations functionalized with a ligand for a receptor that undergoes circadian oscillations could be a suitable strategy to improve the efficacy and safety of a certain therapeutic agent. Therefore, this manuscript aims to review the circadian rhythms in brain barriers, the nanoformulations studied for delivering drugs to the brain, and the nanoformulation with the potential to be conjugated with a chronobiological approach. 

## 2. General Characterization of Brain Barriers

**Figure 1:**
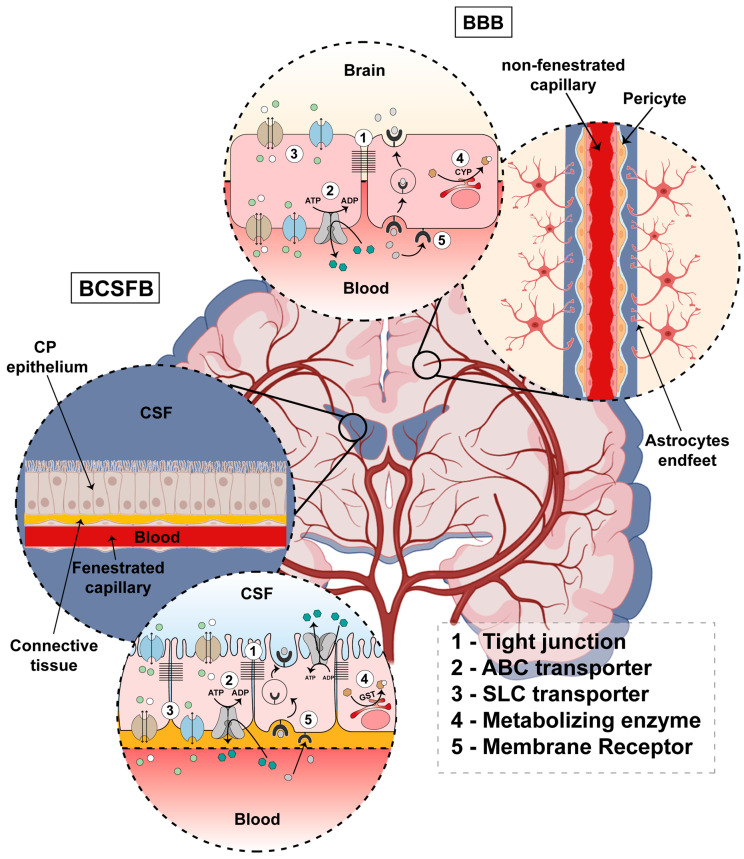
Schematic illustration of the blood–brain barrier (BBB) and blood–cerebrospinal fluid barrier (BCSFB). The BBB is composed of brain capillary endothelial cells, which are sealed by tight junctions and form non-fenestrated capillaries. Pericytes reside in the abluminal side of brain capillaries and incompletely cover the neurovascular tube. Astrocytes’ endfeet are also part of the BBB and almost completely cover the neurovascular tube at the abluminal side. The BCSFB is composed by choroid plexus epithelial cells (CPECs). CPECs are disposed as a monolayer of cuboidal cells, below which resides a highly vascularized connective tissue with fenestrated capillaries. The molecular hallmarks to limit the traffic of substances that brain barriers possess are also represented in the figure. Tight junctions prevent the paracellular passage of substances. ATP-binding cassette (ABC) transporters couple the extrusion of molecules with the hydrolysis of ATP. The solute carrier (SLC) transporters are involved in the transportation of substances by facilitated diffusion or by secondary active transport. The presence of membrane receptors can mediate the endocytosis and the transcytosis of substances across the brain barriers. Finally, the presence of metabolizing enzymes can induce chemical modifications in a variety of substances.

Due to its vital functions, the brain is the most protected organ in the human body. The exchange of substances between the blood and the brain needs to be strictly regulated. For that function, specialized structures are present in the central nervous system. The BBB limits the traffic of substances between the blood and the brain tissue, while the BCSFB limits the exchange of substances between the blood and the CSF. For a barrier to function properly, brain barriers must allow for the passage of substances like nutrients, ions, hormones, and vitamins, and must be impermeable to xenobiotics and noxious compounds. This protective function also accounts for the impermeability of the brain to therapeutic agents. This selectivity of passage is ensured by some molecular characteristics of physiological barriers. The presence of tight junctions, efflux and influx transporters, metabolizing enzymes, and membrane receptors is responsible for traffic selectivity across the brain barriers. A schematic illustration of the BBB and the BCSFB is represented in Figure 1.

### 2.1. Blood–Brain Barrier

The principal component of the BBB (Figure 1) is brain capillary endothelial cells. The brain capillary endothelial cells are sealed by the presence of thigh junctions. Brain capillaries are non-fenestrated and are surrounded by a basement membrane at the abluminal side. Pericytes sit in the basement membrane, incompletely covering the capillary tube. Pericytes are connected to the endothelial cells via gap junctions and peg-and-socket interactions mediated by N-cadherin. Astrocytes’ endfeet almost completely cover the capillary tube [3,16,17,18]. Pericytes and astrocytes are important regulators of the BBB functions ensured by the brain endothelial cells [19,20,21]. 

In terms of impermeability, tight junctions impair the paracellular movement of molecules. Claudins are one of the types of protein that belongs to thigh junction complexes and are responsible for paracellular sealing [22,23]. Claudins can be divided into two main groups. Barrier-forming claudins are associated with an increase in transendothelial resistance, while pore-forming claudins are associated with a decrease in transendothelial resistance due to their permeability to ions [24]. The most expressed claudin in brain capillaries is claudin-5 [25]. Claudin-5 is a barrier-forming claudin cited as the gatekeeper of the central nervous system [26]. In contrast to what occurred in wild-type mice, the BBB was permeable to small molecules in knock-out mice for the claudin-5 gene [27]. The brain capillary endothelial cells at their luminal surface express ATP-binding cassette (ABC) transporters responsible for the efflux of hydrophobic molecules back into the bloodstream. Among the ABC transporters involved in the efflux of xenobiotics and drugs, ABCB1, ABCC4, ABCC5, and ABCG2 are all expressed in the luminal side of brain endothelial cells [28,29,30,31]. The expression of drug-metabolizing enzymes is also a characteristic of physiologic barriers. Cytochrome P450 (CYP) 1B1 and CYP2U1 are the most expressed CYP450 family members in the brain capillaries [32]. The expression of CYP3A4, which metabolizes nearly of 50% of the available drugs [33], was not detected in brain capillaries [32]. In addition to CYP450 enzymes, protein expression of several isoforms of aldehyde dehydrogenase, glutathione S-transferase, and carbonyl reductase, as well as epoxide hydrolase 1, was detected in brain capillaries [34]. 

Solute carrier (SLC) transporters and membrane receptors can mediate and participate in the uptake of substances by the brain. Brain capillaries express a wide array of SLC transporters. The SLC transporters expressed in the BBB are widely documented in the following citation [35]. SLC family membranes transport a wide array of chemically different substances. Some examples are amino acids, sugars, nucleotides, and hormones [35]. Relative to the endocytic route, the brain epithelium is characterized by low vesicular traffic. Although some membrane molecules like insulin and transferrin (Tf) receptors are present in the brain endothelial cells, these receptors are involved in receptor-mediated transcitotic and endocitotic events [14,36]. 

### 2.2. Blood–Cerebrospinal Fluid Barrier

The BCSFB (Figure 1) is composed by choroid plexus epithelial cells (CPECs), which are bound together by tight junctions. A choroid plexus (CP) is located in each of the four brain ventricles, forming an interface between the blood and CSF. Each CP is composed of a monolayer of cuboidal epithelial cells that rest in a basement membrane. Below the basement membrane resides a network of fenestrated capillaries surrounded by connective tissue rich in fibroblasts and immune system cells [37,38,39]. 

In addition to the barrier function, the CP is responsible for the majority of CSF secretion. CSF secretion involves the active transport of Na^+^, Cl^−^, and HCO_3_^−^ to CSF. The transport of these ions creates an osmotic gradient causing the movement of water toward the brain ventricle [37,40,41]. 

Relative to barrier functions, CPECs are closely bound together by the expression of apical tight junctions. Claudins-1, -2, and -3 are the most expressed claudins in the CP [37]. Claudins 1 and 3 are barrier-forming claudins, while claudin 2 is a pore-forming claudin selective for cations [24]. In addition to the permeability of cations like K^+^ and Na^+^, the expression of claudin-2 was also reported to enhance the paracellular permeability of water [42]. Like brain endothelial cells, CPECs also express ABC drug transporters. ABCC1 and ABCC4 were found to be expressed in the basolateral side of the membrane of CPECs [43,44,45]. Data from rodents have shown an apical localization of ABCG2 and a subapical localization of ABCB1 in CPECs [45,46]. Moreover, in addition to the presence of ABC drug transporters, the CP also shows marked glutathione-s-transferase and sulfotransferase activity [37]. 

Besides the impermeability of deleterious compounds, BCSFB needs to be permeable to some ions and molecules necessary for brain function. Many SLC transporters, which are normally associated with the influx of substances, are expressed in CPECs [47]. Several of them are associated with the transport of ions, sugar, amino acids, vitamins, and hormones [48,49,50,51,52]. Also, in the BCSFB, several receptors involved in the transportation of substances are expressed in the CP. Some examples are the Tf, insulin, and low-density lipoprotein (LDL) receptors, as well as the folate receptor α (FRα), which, in the brain, is specifically expressed in the CP [53].

## 3. Mechanisms of Transport in Brain Barriers

**Figure 2:**
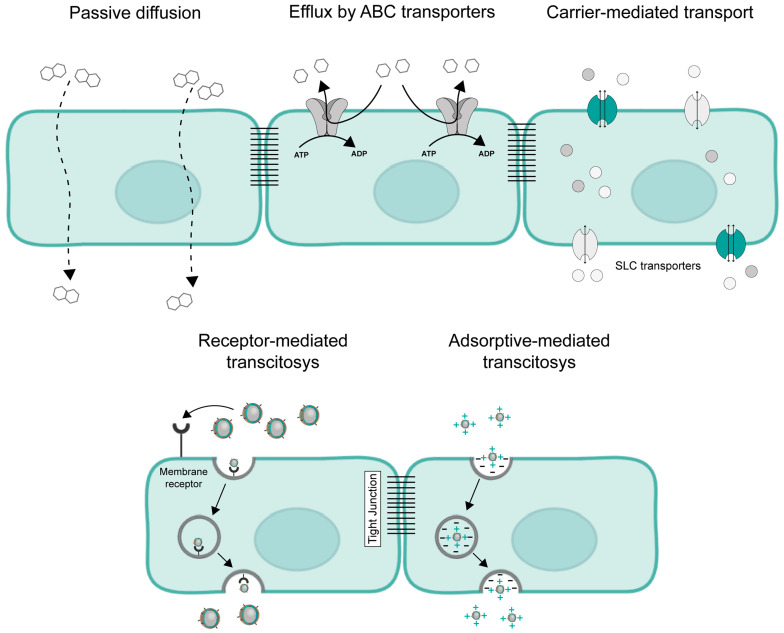
Mechanisms of transport across brain barriers. Small hydrophobic molecules can pass the brain barriers by passive diffusion. The ATP-binding (ABC) cassette transporters transport a great variety of xenobiotics and therapeutic drugs. ABC transporters couple the extrusion of hydrophobic molecules with the hydrolysis of ATP. The solute carrier (SLC) transporters are responsible for carrier-mediated transport. SLC transporters are very heterogenous in structure and function and transport substances by facilitative or secondary-active transport. Receptor-mediated transcytosis involves the binding of a membrane receptor with the subsequent formation of an endocytic vesicle which later fuses with the opposite site of the plasma membrane to release the cargo. In the blood–brain barrier, positively charged substances can pass across the endothelial cells by adsorptive-mediated transcytosis due to the negative charge at the surface of the plasma membrane.

Passive diffusion involves the passage of a substance across the plasma membrane by dissolution in the phospholipid bilayer. This mechanism does not involve a membrane protein, and the direction of the transport is according to a concentration gradient. Passive diffusion is a non-specific mechanism of transport, where the transfer rate is limited by the size and hydrophobicity of the molecules. Only small and hydrophobic molecules are able to pass the brain barriers by passive diffusion. In the case of the BBB, molecules should have a partition coefficient (LogP) between 1.5 and 2.5 and a molecular size of less than 400/500 Da [54]. In addition to the limitations of size and lipidic solubility, passive diffusion is also limited due to the presence of ABC transporters in the plasma membrane, as well as by the presence of metabolizing enzymes (Figure 2).

The efflux transport by ABC transporters is also a hallmark of brain barriers. ABC transporters actively efflux substances out of the cells by primary active transport. They couple the efflux of a substance against the electrochemical gradient with the hydrolysis of ATP [55]. ABC transporters are capable of transporting a great variety of endogenous and exogenous substances. Relatively to therapeutic agents, ABC transporters can transport antidepressants, antiemetics, antiepileptics, antipsychotics, antivirals, antibacterial agents, and many other drugs belonging to other classes [56]. Contrary to the BBB, where the ABC transporters are expressed in the blood-faced side of the plasma membrane [28,29,30,31], data from rodents show a luminal expression of ABCG2 in the CPECs [46], indicating that an efflux back to the CSF can also occur. 

Brain barriers also have to be permeable to some biologically active molecules. SLC transporters, responsible for the carrier-mediated transport mechanism, are widely associated with the transport of substances by brain barriers to supply nutrients to the brain tissue. This makes the SLC transporters a suitable target for drug development. In their vast majority, SLC family members are facilitative or secondary-active transporters. The secondary active transport relies on ion gradients to transport substrates against their electrochemical gradient [57]. For carrier-mediated transcytosis, a substance needs to circumvent brain barriers through influx at the blood-facing membrane and efflux at the opposite side of the plasma membrane from the brain barrier’s epithelial or endothelial cells. The SLC transporters are very heterogenous in structure and range from highly selective transporters to transporters capable of carrying a great diversity of chemically distinct molecules [57,58]. Sugars, amino acids, nucleotides, organic ions, vitamins, and hormones constitute several classes of substrates transported by the carrier-mediated mechanism [57]. Several drugs have been identified to be transported by SLC family members. The antiparkinsonian agent L-DOPA and the anticonvulsant gabapentin are substrates of the large neutral amino acids transporter 1 (LAT1) [59,60]. LAT1 is a heterodimeric transporter formed by SLC7A5 and SLC3A2 and is found to be expressed in the brain barriers [51,61,62]. Moreover, members of the SLC22 and SLCO subfamilies are widely expressed in brain barriers and are greatly associated with drug transportation [63,64]. The conjugation of nanocarriers with substrates of SLC transporters constitutes a nanotechnological approach to study the delivery of drugs through a carrier-mediated mechanism [65].

Receptor-mediated transcytosis is the most specific transport mechanism due to the binding of specific ligands to its membrane receptor. Large molecules, like insulin and lipoproteins, cross brain barriers through this transport mechanism. Receptor-mediated transcytosis involves the binding of a molecule to a specific membrane receptor with consequent endocytosis of the ligand-receptor complex, the trafficking of the vesicle through the endosomal complex, and the fusion of the endocytic vesicle with the opposite side of the plasma membrane [53]. The endocytic vesicle, intracellularly, could also fuse with the lysosomes, and the molecular cargo would be released in the cytoplasm. When developing nanocarriers conjugated with a ligand specific for a receptor, the fusion of the endocytic vesicle with the lysosome must be avoided [66]. Brain capillary cells express membrane receptors involved in the transport of molecules. The most studied ones are the transferrin, insulin, and LDL receptors [14]. These receptors are also among those with potential interest for delivering drugs across the BCSFB [53]. In the BCSFB, FRα could also be interesting because, in the brain, FRα is only expressed in the CP. Besides receptor-mediated transcytosis, membrane receptors could also participate in the hybrid mechanism. For example, in the BCSFB, endocytosed folates released in the cytoplasm can be transported across the apical membrane by the reduced folate carrier [53]. Moreover, a basal-to-apical transport of folates and FRα with a release at the CSF side of the plasma membrane via exosomes was also reported [67]. 

In the BBB, adsorptive-mediated transcytosis was also identified. Adsorptive-mediated transcytosis is mediated by the electrostatic interaction of positively charged substances with the negatively charged molecules in the cell surface at physiological pH [68,69,70]. After the interaction at the luminal surfaces of brain endothelial cells, an endocytic vesicle is formed. The negative charge of the opposite side of the plasma membrane could facilitate the exocytosis of the positively charged molecule [68]. This mechanism is possible due to the presence of sialic acid glycoconjugates and heparan sulfate proteoglycans at the luminal side, and the presence of chondroitin and heparan sulfate-rich proteoglycans at the abluminal side, of the brain endothelial cells [70]. 

## 4. Circadian Rhythms in Brain Barriers

### 4.1. Circadian Rhythms in the Blood–Brain Barrier

There are only a few studies of circadian rhythms in the BBB. The available studies are summarized in Table 1. The molecular clock of pericytes controls BBB paracellular permeability. In brain-specific Bmal1 knock-out mice, an increase in BBB permeability to Evan blue and biotin was reported. The knock-out of Bmal1 in the pericyte cell line TR-PCT1 downregulated the expression of platelet-derived growth factor receptor (PDGFR) β [71]. PDGFRβ functions as a receptor for platelet-derived growth factor (PDGF) BB, which is secreted by the endothelial cells. The signaling between these two molecules is important for BBB integrity [20]. Also, there are available data reporting that BBB permeability to Evan blue was not significantly different between Bmal1 knock-out mice and their littermate controls [72].

A functional molecular clock in the BBB was recently discovered. The circadian clock genes *Bmal1*, nuclear receptor subfamily 1 group D member 1 (*Nr1d1*), Per2, albumin D-box binding protein (*Dbp*), Hepatic leukemia factor (*Hlf*), and thyrotroph embryonic factor (*Tef*) are rhythmically expressed in mouse brain endothelial cells. That rhythmicity is lost in mice with a specific deletion of the endothelial Bmal1 gene [11]. Bmal1 was also reported to be expressed in a circadian manner in the brain microvessels of Wistar rats [73].

Regarding the efflux function at the BBB, the limited available evidence is focused on the ABCB1 efflux transporter. Wistar rats showed a higher brain permeability to MC225, an ABCB1 substrate, at Zeitgeber time (ZT) 15. The peak is coincident with the rodents’ active phase [74]. Specific deletion of the Bmal1 gene in mice endothelial cells abrogated the oscillation in ABCB1-mediated Rhodamine123 efflux [75]. The same results were obtained for Rhodamine B. However, the expression of Abcb1 genes was not rhythmic either in the control mice or in mice with a deletion of endothelial Bmal1 [11]. The protein expression of mouse ABCB1 in the brain capillaries showed no significant differences between ZT 0 and ZTs 6, 12, and 18 [76]. Moreover, in vitro experiments with the human cerebral microvascular endothelial cell line (hCMED/D3) suggest that the circadian oscillations in ABCB1 functionality may possibly be driven by rhythms in Mg^2+^ levels. Mg^2+^ acts as a cofactor for ABC transporters. The rhythmic levels of Mg^2+^ in hCMED/D3 are mediated by the transient receptor potential cation channel (TRPM7), the gene expression of which is directly modulated by the molecular clock [11]. The circadian regulation of efflux activity by the rhythmicity of Mg^2+^ levels was also observed in experiments with Drosophila [77]. 

Still, in ABC transporters, a microarray experiment that contemplated various members of the ABCA, B, C, and G subfamilies reported that only *Abcg2* was expressed in a circadian manner in mouse brain endothelial cells. For the various members of the ABCC subfamily, also known as multidrug resistance proteins, none of the tested transcripts showed daily rhythmicity in their expression [11]. Through a quantitative proteomic analysis, the expression in mouse brain capillaries of a vast array of proteins involved in BBB barrier function was compared between ZT 0 and ZT 6, 12, and 18. The expression of ABCB1, ABCC4, ABCC9, and ABCG2 showed no significant differences between ZT 0 and the other ZTs tested [76]. 

In the case of SLC transporters, *Slc7a5*, which encodes for the large neutral amino acid transporter 1 small subunit 1, showed a circadian rhythm in mouse brain endothelial cells [11]. LAT1 is capable of transporting the therapeutic agents L-DOPA and Gabapentin [59,60]. The rhythmic expression of *Slc7a5* could possibly contribute to circadian oscillations in brain permeability to large neutral amino acids. A study with Sprague–Dawley rats reported significant differences in brain permeability between two time points of all large neutral amino acids (LNAAs), except for leucine and isoleucine [78]. However, according to the proteomic quantitative assay performed by Ogata et al., in mouse brain capillaries, no differences in protein expression between ZT0 and ZT6, 12, and 18 for SLC7A5 and SLC3A2 were reported [76]. SLC3A2 constitutes the heavy subunit of LAT1 [62]. Moreover, the brain levels of total LNAAs were not different between two distinct time points [78]. These data may comprise the possible involvement of LAT1 in the daily variation of permeability to LNAAs. Also, among the SLC proteins tested in mouse brain capillaries, only SLC9A3R2 showed significant differences between ZT0 and ZT 6 and ZT0 and ZT 18 [76]. SLC9A3R2 is a regulatory protein that interacts with the sodium/hydrogen exchanger (NHE) 3 [79]. The protein expression of some important SLC transporters, like the glucose transporter (GLUT) 1, the organic anion transporter (OAT) 3, monocarboxylate transporter (MCT)1, and MCT8, showed no daily significant differences in mouse brain endothelial cells [76].

Relative to receptors involved in receptor-mediated transcytosis, the protein expression of insulin and transferrin receptors does not show any significant differences between ZT0 and ZT6, 12, and 18 [76]. The insulin and transferrin receptors are among the most studied targets to circumvent the BBB to deliver drugs into the brain tissue [14]. Data from rodents show that sleep restriction increased the vesicular traffic across the BBB [80]. The brain influx of human recombinant interleukin (IL) 1α was different between two timepoints (8 h vs. 24 h) [81]. Data suggest that the transport of IL-1β across the BBB is mediated by a mechanism dependent on the type II IL-1 receptor [82]. However, no circadian oscillations were observed for this receptor. Amyloid β (Aβ) levels showed circadian oscillations in mouse brain interstitial fluid [83]. These oscillations may be due to circadian rhythms in the processes of production and/or elimination of Aβ from the brain. In the BBB, among the receptors and transporters involved in the transport of Aβ, circadian rhythms were only found for ABCB1 at the functional level and for *Abcg2* at the gene expression level [11].

Finally, regarding molecules involved in paracellular permeability, in mouse brain capillaries, no significant differences in protein expression for claudin 5, occludin, and tight junction protein 1 between ZT0 and ZT6, 12, and 18 were observed [76].

### 4.2. Circadian Rhythms in the Blood–Cerebrospinal Fluid Barrier

The available data of circadian rhythms in the BCSFB is summarized in Table 2. The CP harbors a functional circadian clock dependent on sex. Quintela et al. firstly showed a rhythmic expression of *Bmal1*, *Cry2*, and *Per2* in the CP of female rats, and a rhythmic expression of *Cry2* and *Per2* in the CP of male rats [10]. However, there are some reports of rhythmic expression of the *Bmal1* gene in the CP of male rats [73,84]. Furthermore, a higher expression of *Bmal1* during the dark phase when compared with the expression during the light phase in the CP of male rats was also reported [85]. A phase advance of 6 h in the rhythmicity of *Bmal1* and the abolishment of a *Per2* rhythm expression were observed in the CP of ovariectomized rats when compared to the sham-operated controls [9]. Moreover, microarray studies found sex-related differences between sham-operated, ovariectomized, and orchiectomized rats in the expression of clock genes in the CP. *Bmal1* showed higher expression in the CP of female rats, while the circadian-associated repressor of transcription (*Ciart*), *Dbp*, *Per2*, and *Per3* were shown to be more highly expressed in the CP of male rats [4,86]. *Bmal1* was downregulated and *Ciart*, *Dbp*, *Per2*, and *Per3* were upregulated in ovariectomized rats when compared with their sham-operated counterparts [4,87]. Orchidectomy of male rats upregulated the expression of *Bmal1* and downregulated the expression of *Ciart*, *Dbp*, *Per2*, and *Per3* [4,87]. Despite the downregulation of Bmal1 and the unaffected expression of *Per1* upon ovariectomy [4,87], 17β-estradiol (E2) treatment of rat CPEC upregulated the expression of the Bmal1, Per1, and Per2 genes. The effect observed in *Per1* and *Per2* was mediated by the nuclear estrogen receptors [9].

The CP molecular clock showed robust rhythms and higher single-cell synchrony than the suprachiasmatic nucleus (SCN) circadian clock [88]. However, the intensity of rhythms in the CP, contrary to what occurs in SCN, is not recovered after exposure to the anesthetic sevoflurane [89]. The robustness of the rhythms in the CP clock is due to the coupling effect mediated by gap junction communication between the CPECs [88]. The adrenalectomy of adult male Wistar rats abolished the rhythm in Per1 expression and decreased the robustness of the *Per2*, *Nd1r1*, and *Bmal1* rhythms in the CP. Moreover, it was shown that dexamethasone upregulates Per1 expression in CP in vivo and influences PER2 turnover in mice CP explants. The presence of mifepristone, a glucocorticoid receptor blocker, blocked the effect of dexamethasone in PER2-driven bioluminescence rhythms in mice CP explants as well [84]. Therefore, according to the available data, the CP harbors a functional molecular clock with robust rhythms and is entrained by estrogens and glucocorticoids.

Circadian rhythms in CP functions were extensively reviewed in the following reference [90]. As an “active interface”, the uptake of Aβ was rhythmic in the human epithelial CP papilloma cell line (HIBCPP) [91]. These data may account for the circadian oscillations of Aβ levels in mouse brain interstitial fluid [83]. In addition to the rhythmic uptake of Aβ in a CP cell line, circadian rhythms were also found in the Aβ scavengers’ expression in the CP. Some of them were ligands for specific receptors. Regarding Aβ scavengers, apolipoprotein J (*ApoJ*), which gradually reduces Aβ aggregation and accumulation [92,93], was shown to be rhythmically expressed in the CP of female rats, with a peak during the dark phase. The sex-dependent rhythm was independent of the female sex hormone background [94]. Transthyretin (*Ttr*), which is capable of preventing Aβ aggregation and disrupts Aβ fibrils [95,96], was reported as being sex-independently rhythmically expressed in rat CP with a peak during ZT 16 [94]. As the active period of rats is during the dark phase, the peak times of *ApoJ* and *Ttr* circadian expression are not concordant with the enhanced clearance of Aβ from the CSF to the blood during the sleep phase [97]. The CP circadian clock was affected by age and Aβ deposition in the Alzheimer’s disease animal model consisting of APP/PS1 mice [98]. Aralkylamine N-acetyltransferase, the enzyme responsible for the circadian synthesis of melatonin, presented rhythmic expression in rat CP. However, the secretion of melatonin by porcine CP explants did not follow a rhythmic pattern [99]. Moreover, an investigation with APP/PS1 mice showed rhythmic *Ttr* expression in the CP of the wild-type 12-month-old female mice. For the angiotensin-converting enzyme, which is capable of cleaving Aβ, the same pattern was observed for 6-month-old wild-type female mice [91]. 

ABC drug transporters are a great component of brain barriers. As previously described, data exist regarding daily variations in the brain distribution of some substances. Circadian rhythms in efflux transporters may explain those daily variations. *Abcc4* was shown to be rhythmically expressed in the rat CP and in the HIBCPP cell line. Also, ABCC4 may be partially responsible for the circadian transport of methotrexate across the HIBCPP cells [12]. *Abcc1* and *Abcg2* have sex-dependent circadian expression in rat CP. *Abcc1* was rhythmically expressed in the CP of male rats and *Abcg2* in the CP of female rats [12]. Regarding the circadian rhythms of SLC transporters, detoxification enzymes, and membrane receptors, the evidence available is scarce. Among the SLC drug transporters expressed in the CP, which are briefly summarized in [63,64], there is only one report of *Slc22a8*, which encodes the organic anion transporter 3, having a circadian expression in the CP [12]. Regarding paracellular permeability, data show that the expression of several tight junctions is affected by the photoperiod [100].

**Table 2:** Summary of the studies involving circadian rhythms in the blood–cerebrospinal fluid barrier (BCSFB).

Topic	Animals/Cell Model	Results	References
Clock genes	Proestrus adult female and male Wistar rats	The choroid plexus molecular clock is dependent on sex. Cryptochrome (*Cry*) 2 and period (*Per*) 2 showed rhythmic expression in the choroid plexus of female and male rats. The expression of brain and muscle ARNT-like 1 (*Bmal1*) was only rhythmic in the choroid plexus of female rats.	[10]
	Primary culture of rat choroid plexus epithelial cells	*Bmal1*, circadian locomotor output cycles kaput (*Clock*), and *Per2* were shown to be rhythmically expressed in choroid plexus epithelial cells. For *Per1* and *Cry2*, no significat rhythmic expression was found.	[10]
	Male Wistar rats	Bmal1 gene showed rhythmic expression in the rat choroid plexus.	[73]
	Per2::dLuc transgenic male rats on a Wistar rat background	At the lateral ventricle choroid plexus, *Bmal1* expression was higher at zeitgeber time (ZT) 22 than ZT10, and the expression of *Per1* and *Per2* was higher at ZT10 than ZT4. At the fourth-ventricle choroid plexus, *Bmal1* expression was higher at ZT 22 than ZT10, the expression of *Per1* was higher at ZT10 than ZT4, and the expression of *Per2* was higher at ZT16 than ZT4.	[85]
	Female Wistar rats	The abolishment of the rhythmic expression of *Per2* and a phase advance of 6 h in *Bmal1* expression were reported in the choroid plexus of ovariectomized rats.	[9]
	Primary culture of Wistar rat choroid plexus epithelial cells	The treatment of primary rat choroid plexus epithelial cells with 17β-estradiol (E2) led to the upregulation of the *Bmal1*, *Per1*, and *Per2* genes. The E2 effect observed for *Per1* and *Per2* was mediated by the nuclear estrogen receptors.	[9]
	Rats	*Bmal1* showed a higher expression in the choroid plexus of female rats when compared to male rats. Circadian-associated repressor of transcription (*Ciart*), albumin D-box binding protein (*Dbp*), *Per2*, and *Per3* were shown to be more highly expressed in the choroid plexus of male rats.	[4,86]
	Wistar rats	*Bmal1* was downregulated, and *Ciart*, *Dbp*, *Per2*, and *Per3* were upregulated, in the choroid plexus of ovariectomized rats. *Bmal1* was upregulated, and *Ciart*, *Dbp*, *Per2*, and *Per3* were downregulated, in the choroid plexus of orchidectomized rats.	[4,87]
Molecular clock robustness	Per2::*Luc* transgenic mice under C57BL/6J background	The molecular clock in the choroid plexus showed more robust rhythms than the suprachiasmatic nucleus molecular clock. The rhythms were evaluated by the Per2-driven luciferase rhythms of explants removed from Per2::*Luc* transgenic mice.	[88]
	Cultured choroid plexuses of Per2::*Luc* transgenic mice	The choroid plexus showed higher single-cell synchrony in Per2-driven luciferase rhythms than in the suprachiasmatic nucleus. The treatment of the cultured choroid plexuses with a gap junction blocker dose dependently decreases the amplitude, increases the period, and decreases the cell synchrony in Per2-driven luciferase rhythms.	[88]
	Per2::*Luc* transgenic male rats	The intensity of rhythms in the CP, contrary to what happens in SCN, is not recovered after exposure to sevoflurane.	[89]
Entrainment by glucocorticoids	Male Wistar rats	*Per1* rhythmic expression was abolished and a decrease in the robustness of *Per2*, nuclear receptor subfamily 1 group D member 1 (*Nd1r1*), and *Bmal1* rhythms in the choroid plexus was observed in adrenalectomized adult male Wistar rats. The treatment with dexamethasone upregulated *Per1* expression and increased the amplitudes of rhythms in the expression of *Bmal*, *Per2*, and *Nr1d1* in the male Wistar rat choroid plexus in adrenalectomized rats.	[84]
	Per2::*Luc* mice choroid plexus explants	Dexamethasone influenced PER2 turnover in mouse choroid plexus explants. DEX treatment increased the amplitude and caused a phase shift in PER2-driven bioluminescence rhythms in mouse choroid plexus explants. The glucocorticoid receptor blocker mifepristone blocked the dexamethasone-induced phase shifts in PER2-driven bioluminescence rhythms in mouse choroid plexus explants. The induction of phase shifts in the choroid plexus clock is partially mediated by the activation of protein kinase A and the activation of the MAP kinase pathway.	[84]
Amyloid β uptake, scavengers, and the influence on molecular clock	Human epithelial choroid plexus papilloma (HIBCPP) cell line	Amyloid β uptake was rhythmic in the human choroid plexus papilloma HIBCPP cell line.	[91]
	Wistar Han Rats	Transthyretin showed rhythmic expression in the rat choroid plexus, with a peak during ZT16. Apolipoprotein J presented rhythmic expression in the choroid plexus of female rats, with a peak during the dark phase. The rhythmic expression of Apolipoprotein J was independent from a female sex-hormone background.	[94]
	APP/PS1 mice (Alzheimer’s disease mice model)	They compared the daily expression of amyloid β scavengers in 6- and 12-month APP/PS1 (Alzheimer’s disease mice model) and wild-type mice. Transthyretin showed a rhythmic expression in wild-type 12-month-old female mice. The angiotensin-converting enzyme was rhythmic in the choroid plexus of 6-month-old wild-type female mice.	[91]
	APP/PS1 mice (Alzheimer’s disease mice model)	*Bmal1*, *Per2* and *Cry2* were rhythmically expressed in the choroid plexus of 6- and 12-month-old APP/PS1 and wild-type mice. In female and male mice 12 months of age, *Bmal1* was shown to be rhythmically expressed only in wild-type mice, but not in APP/PS1 mice. *Cry2* only showed rhythmic expression in wild-type 6-month-old mice when compared with correspondent-aged APP/PS1 mice. In male mice 6 months of age, *Per2* expression was rhythmic only in APP/PS1 mice, and not in wild-type mice.	[98]
ATP-binding cassette (ABC) transporters	Wistar rats	*Abcc4* was reported to be rhythmically expressed in the rat choroid plexus. *Abcc1* and *Abcg2* circadian expression in the choroid was found to be dependent on sex. *Abcc1* showed rhythmic expression in the choroid plexus of male rats. *Abcg2* showed rhythmic expression in the choroid plexus of female rats.	[12]
	HIBCPP cell line	*Abcc4* was shown to be rhythmically expressed in the HIBCPP cell line. ABCC4 may be partially responsible for the circadian transport of methotrexate across the HIBCPP cell line.	[12]
Solute carrier (SLC) transporters	Wistar rats	The organic anion transporter 3 encoding gene, *Slc22a8*, was shown to be rhythmically expressed in the rat choroid plexus.	[12]
Melatonin production	Wistar rats	The aralkylamine N-acetyltransferase showed circadian expression in the rat choroid plexus.	[99]
	Porcine CP explants	The melatonin secretion by porcine CP explant did not show a circadian pattern.	[99]

## 5. Nanoformulations as Therapy for Central Nervous System Diseases

Diseases affecting the nervous system represent a worldwide public health problem, particularly neurodegenerative diseases such as Parkinson’s (PD) and Alzheimer’s disease (AD), stroke, multiple sclerosis, and dementia [101]. These might result from different causes, including genetic disorders; neurodegeneration and inflammation; lifestyle; structural, biochemical, or electrical abnormalities in the brain; and traumatic brain injury [102]. 

The major challenge of treating these conditions results from the selective permeability of brain barriers, namely, the BBB, that limit most drug molecules from reaching their therapeutic targets in the brain. This blockage is due to the presence of active efflux systems and tight junction overexpression, of which ABC protein superfamily transporters, such as the ABCB1, are the most effective in removing drugs from the brain and pumping them back into the blood [103].

As a consequence, higher doses are required, causing toxic effects and limiting the treatment effectiveness of central nervous system (CNS) diseases [104]. In recent years, several strategies have been developed to enhance drug delivery to the brain, with some being more beneficial and minimally invasive. They include osmotic disruption of the tight junctions, chemical modification of drugs, ultrasound-mediated BBB opening, intrathecal therapies, local delivery by neurosurgery, and nanoparticle mediation. However, some of these approaches are risky, as they damage and compromise the integrity of the membranes, causing the influx of unwanted molecules in the CNS. Among these, nanoparticle-based approaches are emerging as effective delivery systems. Nanoparticles (NPs) are nanoscale particles ranging from 1 nm to 1000 nm in diameter, employed to encapsulate therapeutic agents within or conjugate onto their surfaces [105]. Its benefits include the capability of creating small and more stable structures to encapsulate poorly soluble drugs, prolonging their circulation time, improving their bioavailability, and controlling the release rate. Additionally, NP surfaces can be functionalized with specific moieties for targeted delivery to a region of interest [106]. Indeed, one of the main advantages is the potential to optimize nanoparticle design through size, shape, and surface modifications to improve interaction and diffusion through cells and tissues. There is also the possibility of conjugating two or more therapeutic compounds for combination therapy, including nucleic acids, proteins, peptides, antibodies, and anticancer drugs [107]. 

For brain targeting, some requirements must be met: non-toxicity, small size, biocompatibility, high targeting efficiency, long and stable blood circulation, favorable pharmacokinetic properties, and biodegradability. Ideally, these nanosystems should also be easy to produce, cost-effective, and easy to scale up [108]. Diverse nanocarriers have been explored based on the method of preparation, type of materials used, drug loading, and release behavior. Special attention has been given to liposomes, micelles, inorganic nanoparticles, polymeric and dendrimers-based nanoparticles, exosomes, quantum dots, and carbon nanotubes [109,110,111]. 

In the same way, various methods and routes of delivery have been investigated for their ability to enhance the efficiency of delivering nanoparticles to the brain and allowing them passage through the brain barriers. In the following section, we discuss the promising and recent developments in new nanoformulations as forms of therapy for CNS diseases.

### 5.1. Polymeric NPs

Polymeric nanoparticles are solid colloidal particles made of polymers of natural or synthetic origin. With their versatile nature, polymers can vary in composition, combining two or more different materials to create hybrid nanosystems (lipid–polymer hybrids, metal–polymer hybrids, etc.) [112]. Compared to other carriers, they possess higher physical stability when in contact with biological fluid, which is beneficial for sustained drug release [113]. Moreover, the large surface area potential for functionalization makes them very promising carriers in terms of improving drug delivery across the BBB. Polymeric NPs are efficiently transported across endothelial cells via receptor-mediated, carrier-mediated, and adsorptive-mediated pathways [114].

For example, a number of chemotherapeutic drugs have been encapsulated in nanoparticles composed of chitosan, poly (lactic-co-glycolic) acid (PLGA), polyethylene glycol (PEG), dextran, and dendrimers [115]; however, polymeric NPs present some limitations for application in brain tumors due to their insufficient retention and accumulation within the tumors [116]. Coating the surface of NPs with ligands or stabilizers has shown promising results in terms of targeting the therapeutic molecules to specific receptor-targeted sites via receptor-mediated endocytosis. In light of this, ligands like Tf, apolipoprotein E, apolipoprotein B, apolipoprotein A, and cell-penetrating peptides have been studied [117,118]. Recently, Nabi et al. [119] developed chitosan NPs conjugated with Tf for the delivery of riluzole, which is used to treat amyotrophic lateral sclerosis, and demonstrated the enhanced brain delivery of Tf-conjugated NPs in comparison to the NPs without the ligand. Another research group demonstrated a 1.9-fold increase in cellular uptake of 1, 2-Distearoyl-sn-glycero-3-phosphoethanolamine (DSPE)-Apo-E-modified solid lipid NPs [120]. The addition of stabilizers, such as PEG and Polysorbate 80, a non-ionic surfactant, prolong NPs’ circulation time, reduce the adhesion between the particles, and reduce their clearance [121]. Polysorbate 80 has been conjugated with PLGA to improve the permeability of acetylpuerarin across the BBB and enhance its brain-protective effects in rats [122]. Di Mauro and co-workers investigated the functionalization of polyester-based NPs with two different peptides, AGBBB015F and Regulon, for the delivery of paclitaxel. These peptides possess an affinity for the low-density lipoprotein receptor (LDLR), improving brain permeability in comparison to non-functionalized NPs in the U-87 MG cell line [123]. Lastly, dendrimers are three-dimensional branched polymers with a unique structure and properties, such as small size (typically lower than 50 nm), well-defined shape, monodispersity, and multivalent surface reactive groups suitable for the incorporation of targeting ligands [101]. The potential for brain drug delivery of a lower-generation polyamidoamine (PAMAM) dendrimer loaded with memantine was recently accessed in vitro and in vivo in an Alzheimer’s disease-induced mouse model [124]. This preliminary study reported a favorable effect of memantine-encapsulated PAMAM-lactoferrin conjugate on the cognitive behavior of Alzheimer’s disease-induced animals. 

### 5.2. Metallic NPS

Metallic nanoparticles (MNPs) have been extensively applied in imaging and drug delivery for various therapeutic agents. They are formed from metal cores composed of pure metals or metal oxide (e.g., silver, gold, palladium, titanium, zinc, copper, gadolinium, iron oxide, hydroxides, sulfides, phosphates, etc.), usually covered by a shell made of organic or inorganic materials [125,126]. Their great tunable optical properties, small size, facility to infiltrate into biological membranes, serum stability, and long half-life are some of their finest characteristics for biomedical applications [127]. Their smaller size in comparison to polymer and lipid NPs offers an advantage in terms of the crossing of brain barriers. However, due to their dense structure, therapeutic agents have to be conjugated to the surfaces of the NPs [128]. MNPs’ surface can also be functionalized with targeting ligands through H-bonding, covalent bonding, or electrostatic interactions to alter its pharmacokinetics properties and enhance its specificity to target tissues, allowing treatment to occur at the cellular level [127]. For drug delivery to the brain, peptide-conjugated NPs represent a novel strategy that has shown promising results. In a comparative study conducted by Wang et al. [129], different surface modifications were performed on superparamagnetic iron oxide NPs (SPIONs), and their distribution and diffusion were observed in rat brains after injection into the rat substantia nigra. The prepared nanoparticles were either coated with PEG and maleic anhydride (Mal) (Mal-SPIONs), with bovine serum albumin (BSA) (BSA/Mal-SPIONs), or with Arg–Gly–Asp peptide (RGD) (RGD/Mal-SPIONs). It was observed by transmission electron microscopy that an abundant diffusion of RGD/Mal-SPIONs into the thalamus, frontal cortex, temporal lobe, olfactory bulb, and brain stem occurred after injection. For rats injected with BSA/Mal-SPIONs, only a few NPs diffused to the afore-mentioned brain areas, while Mal-SPIONs were expelled out of the brain. The magnetic properties of metallic NPs are of relevance for brain cancer treatment [130]. The application of an external magnetic field can guide the NPs to precise locations, while photothermal therapy using near-infrared laser can be used to increase the internal temperature of magnetic NPs, producing a hyperthermic effect and resulting in irreversible damage or death of tumor cells. A recent work demonstrated the efficacy of applying an alternative magnetic field in the treatment of C6 glioma in rats [131]. Temozolomide (TMZ)-loaded SPIONs conjugated with folic acid (FA) (TMZ/MNPs-FA) were prepared, and rats were treated with free TMZ, MNPs-FA, and TMZ/MNPs-FA in either the presence or absence of the magnetic field [131]. The strategy of combined therapy could significantly suppress tumor growth, increase survival rate, and promote apoptosis. Nonetheless, extensive research into the long-term toxicity of metal NPs is necessary prior to considering its use in clinical therapy.

### 5.3. Liposomes

Liposomes are self-assembled organic NPs of spherical shape created from natural or synthetic phospholipids and/or cholesterol that organize into a phospholipid bilayer membrane. They can be classified in relation to the number of bilayers and range from 30 nm to several micrometers in size [132,133]. Hydrophilic drugs can be encapsulated in the aqueous core, while hydrophobic and amphiphilic drugs can be embedded within the phospholipid bilayer. Liposomes have been widely investigated as drug vehicles and are probably the most studied nanocarriers due to their low toxic side effects compared to other drug delivery systems, protection of the encapsulated drugs from physical degradation and clearance, ability to deliver both hydrophilic and lipophilic compounds, and easy preparation [134]. Some drawbacks include fast degradation of the phospholipids and inability to provide sustained release of the drugs. Nevertheless, liposomes can also easily be surface-modified to overcome some of these limitations. Given this, some liposomal formulations have been approved by the U.S. Food and Drug administration to be used in clinics [101,135]. Liposomal formulations display similarities to the lipid bilayer of the endothelial cell membrane, which facilitates the crossing of the BBB. The mechanism of transport includes receptor-mediated transcytosis and adsorptive-mediated transcytosis [136]. In relation to brain delivery, a cationic liposome (SGT-53) with anti-transferrin receptor antibody, encapsulating wildtype p53 sequence, has been investigated in clinical trials for the treatment of glioblastoma [132]. The Tf receptor (TfR) has indeed remained a popular target for the brain [137]; however, the safety of TfR-targeting on liposomes was checked after adverse effects after intravenous administration were found, even though targeting and subsequent transport across the BBB were accomplished [138]. Targeting of liposomes with peptides is also attainable. A research group developed doxorubicin (DOX)-loaded liposomal NPs functionalized with a short nontoxic peptide derived from Aβ 1-42 (SP-sLip) (SP-sLip/DOX), which interacts with the lipid-binding domain of exchangeable apolipoproteins achieve brain-targeted delivery [139]. The brain targeting efficiency, distribution, and anti-cancer effect of SP-sLip/DOX were significantly enhanced in relation to doxorubicin-loaded plain liposomes. Another study investigated glutathione (GSH) conjugation to different liposomal formulations and its influence on the brain delivery of methotrexate (MTX) in rats [140]. GSH-PEG liposomal MTX NPs were based on two different phosphatidylcholines, one derived from soy (HSPC) and another from egg yolk (EYPC). As conclusion, GSH-PEG-HSPC liposomes HSPC increased brain delivery of MTX by fourfold (and eightfold compared to free MTX), while GSH coating on PEG-EYPC liposomes did not result in further enhancement of brain uptake, demonstrating that the brain-targeting effect of GSH is highly dependent on the liposomal formulation. 

### 5.4. Carbon Nanotubes

Carbon nanotubes (CNTs) are hollow cylindric molecules composed of rolled-up graphite sheets of single-walled or multi-walled tubes, with diameters in the range of 1 to 100 nm. Each carbon atom establishes covalent bonds with three neighbor atoms in the graphite, forming a repeating pattern of sp2 hybridized carbon atoms in a hexagonal arrangement [141]. This unique structure provides considerable strength to CNTs, offering a high surface area, high aspect ratio, low density, electrical and thermal conductivity, ease of functionalization, and high drug loading capacity, and is considered as a novel delivery carrier [142]. The hollow interior space and high surface area provide enough space for multiple drugs and targeting ligands to be attached, which represents a great advantage of CNTs over other delivery systems for both diagnostic and therapeutic applications. Apart from their promising properties, CNTs have gained particular interest due to their needle-like shape, which is correlated with the ability to penetrate biological membranes [143]. Nonetheless, some concerns have arisen regarding their non-biodegradable state and toxicity [144]. In the past few years, CNTs have also been investigated for brain drug delivery. The ability of functionalized CNTs to cross the BBB and accumulate in the brain has been tested in in vitro and in vivo models after systemic administration [145,146]. You and his group [147] designed a dual-functionalized carbon nanotube for glioma therapy. For that purpose, they constructed multi-walled CNTs decorated with the cell-penetrating peptide transactivating transcriptional activator (TAT), the polymer polyethylenimine (PEI), and a cancer-targeted molecule biotin loaded with oxaliplatin (TBCNT@OXA). The targeting and penetration ability of the nano-construct was tested in an in vitro BBB model. In conclusion, the dual targeting highly improved the permeability of brain endothelial cells over free oxaliplatin. Another research group [148] explored the use of CNTs for the treatment of brain cancer. In this study, a polyethylene glycol-linked conjugate of CNTs with the phytochemical mangiferin (MF) was synthesized (CNT-PEG-MF), and its safety and anticancer activity was investigated in a human glioblastoma astrocytoma cell line (U-87 MG cells). However, the ability to cross brain barriers still needs to be tested. Carbon nanotubes have also been studied for Parkinson’s disease treatment, since the current options are limited. Guo et al. [149] attempted to deliver dopamine (DA) to the brains of mice using single-walled CNTs (SWCNTs) functionalized with PEG and lactoferrin (Lf) (SWCNT-PEGs-Lf), a cationic iron-binding glycoprotein that has previously been proven to facilitate transport across the BBB when conjugated to nanoparticles, thus being considered a brain-targeting ligand [150]. The SWCNT-PEGs-Lf was able to significantly reduce the levels of oxidative stress, tumor necrosis factor-α, and IL-1β in a mouse model of the disease and to increase the density of tyrosine hydroxylase-immunoreactive. Furthermore, a safe dose was established for the application of this CNTs. Again, a dual targeting strategy demonstrated precision in delivering drugs into the brain.

### 5.5. Exosomes

Exosomes differ from synthetic NPs in that they are of natural origin. They are a subgroup of nanosized extracellular vesicles (diameter ∼30–150 nm) enclosed by a lipid bilayer membrane, endogenously produced by most eukaryotic cells and subsequently secreted in the extracellular space by fusion with the cellular membranes. Exosomes act as a route of intercellular communication by transferring lipids, proteins, and RNAs between cells, and participate in a wide variety of physiological and pathological processes. Similarly to liposomes, exosome’s structure enables them to incorporate hydrophobic drugs within the lipid bilayer membrane and hydrophilic drugs into the aqueous core [151], although exosomes have superior biocompatibility, which improves their stability in the bloodstream and their effectiveness in vivo. Despite their great therapeutic potential, their practical application is limited due to the lack of standardized methods for their efficient isolation and purification protocols [152].

As discussed above, synthetic nanocarriers have different drawbacks. In contrast, exosomes possess distinctive properties, in addition to a natural BBB-crossing ability, to function as an efficient drug delivery system to the brain [153]. The mechanisms of exosome entry in the brain include paracytosis, transcytosis, fusion, and ligand–receptor interactions. Nonetheless, surface modification can increase exosome diffusion through the BBB [154]. Recently, Xu Liu and collaborators [155] conjugated exosomes with anti-CD22 monoclonal antibody fragments and DOX to improve their targeting ability to malignant mature B lymphocytes, which express CD22 antigen and reduce the associated toxicity. Targeted exosomes prolonged the life expectancy of tumor-bearing mice and enhanced the pro-apoptotic levels of DOX. Regarding cancer therapy, exosomes have also been explored for glioma treatment [156]. A combined strategy was applied by loading exosomes simultaneously with SPIONs and curcumin and then conjugating the exosome membrane with neuropilin-1-targeted peptide, a transmembrane glycoprotein overexpressed in glioma cells [157]. When applied to orthotopic glioma models, the exosomes were able to cross the BBB, and the combination of SPION-mediated magnetic flow hyperthermia and curcumin-mediated therapy showed a potent synergistic antitumor effect. Neurodegenerative diseases such as Parkinson’s could also benefit from the potential applications of exosomes. Huan Peng et al. constructed a nanocarrier for nasal administration with exosomes derived from mesenchymal stem cells (MSCs), which have a therapeutic effect on PD by themselves, mainly due to the presence of micro RNAs (miRNAs) and proteins [158]. In addition, they utilized curcumin, which can inhibit the aggregation of α-synuclein and reduce the toxicity of aggregates to dopaminergic neurons [159]. Moreover, SPIONs were also assembled to be used as magnetic resonance imaging contrast agents for the purpose of tracking the accumulation of nanocarriers in the brain. The results from the PD mice model revealed an improvement in movement and coordination and suggested good prospects for the use of the developed nanocarrier in the treatment of other neurodegenerative diseases. Exosomes were once considered to be functionless cellular waste; however, with increasing evidence, not only have they been confirmed to play an important role in the body’s physiology, but they also demonstrated great promise as targeted drug delivery vehicles.

## 6. Circadian Rhythms in Nanoformulations for Central Nervous System Penetration

To overtake the difficulty of treating CNS disorders and to reach the brain, actual pharmaceutical therapies use different delivery routes for their purposes: delivery across the BBB, intrathecal delivery, and intranasal delivery [160]. The most studied approach focuses on direct BBB delivery due to its minimally invasive nature [160]. However, there are remarkable troubles preventing drugs from passing through this barrier due to its structure [160]. Nanoformulations have emerged as alternatives to overcome these troubles. The mechanism of uptake appears to be by endocytosis mediated by receptors, followed by transcytosis of the drug-loaded nanoformulations and drug release in the brain or within endothelial cells [161]. Furthermore, little attention has been paid to the influence of daily patterns, such as circadian rhythm, on nanoformulations uptake and passage across the BBB. Nanotechnological strategies have been focused on designing delivery carriers functionalized with ligands targeting proteins of the BBB in order to enhance brain delivery. Receptors such as LDLR, TfR, GLUT1, and MCT1 are highly expressed in the BBB and are common target receptors [162]. In addition, these receptors and transporters have shown rhythms in their expression and function, or their expression has been shown to be regulated by clock genes. Daily variations in the *ldlr* gene in mouse livers were abolished in clock knock-out mice [163]. The promotor region of the human *ldlr* gene possesses three putative E-box sequences, which represent putative sites for binding of the CLOCK:BMAL1 complex [164]. There are also data suggesting that transferrin receptor (TfR) 1 expression is indirectly regulated by the clock genes in tumors derived from the mouse cell line colon-26 [165]. For GLUT1 and MCT1, the existing data point to the possibility that these transporters are not rhythmically expressed in the BBB [76,166]. However, we need to take into account that circadian rhythms are influenced by factors like sex and species, as well as the fact that GLUT1 and MCT1 show rhythms in other tissues or cell lines, and even the *glut1* gene has been shown to be regulated by the neuronal PAS domain protein 2 NAPS2 in a human cell line [167,168,169,170]. These data, together with the point that these receptors and transporters are common targets for nanoformulations, makes the nanocarriers which contain the receptors and transporters addressed here targets with the potential to be tested according to biological rhythm. Below, in Table 3, we describe examples of nanoformulations applied to treat CNS diseases which have the receptors and transporters described above as targets. The use of glucose as a ligand in nanocarriers to enable the active translocation of GLUT1 in BBB has been strategically applied [171]. Anraku et al. developed polymeric micelles functionalized with glucose to be recognized and internalized by GLUT1 on BBB. The results showed preferential and successful crossing of the barrier, and the use of glucose ligand facilitated the transport and accumulation into the brain [172]. Another group developed multiple glucose-modified micelles to deliver antisense oligonucleotides (ASOs) to the brain and treat several CNS disorders. They achieved accumulation in the brain tissue after 1 h of intravenous administration and RNA knockdown in different regions. Magnetic nanoparticles loading ibuprofen and glucose-modified were prepared and characterized for posterior evaluation of brain cells’ uptake capacity [173]. This research group pretended to efficiently deliver ibuprofen through GLUT1 under an external magnetic field. Drug release studies showed a promising strategy to load and deliver drugs to brain. Thus, we hypothesize that a more efficacious treatment would be achieved if researchers were to consider the circadian expression of the GLUT1 transporter and the possibility of nanoformulations being more easily internalized at certain time points of the day. Another example was proposed and developed by Ak et al. They designed cetyl palmitate-based nanoparticles with β-hydroxybutyric acid, a targeting ligand for MCT1, and carmustine and temozolomide for glioblastoma treatment [174]. In vitro studies proved their biocompatibility and antitumor activity. Moreover, more nanoparticles were captured by MCT1 overexpressed brain cells. P-aminophenyl--D-mannopyranoside (MAN) decorated doxorubicin-loaded dendrigraft poly-l-lysine with acid-cleavable Tf coating outside (DD-MCT) was designed to be recognized by the TfR and to be more easily internalized into the brain parenchyma [175]. DD-MCT reached glioma cells and accumulated to a higher extent, improving the therapeutic outcome of cancer. Lam et al. enhanced glioblastoma therapy by developing nanoparticles functionalized with Tf and loading temozolomide and the bromodomain inhibitor JQ1 [176]. Nanoparticles were capable of traversing the BBB in mice, as well as reaching tumor cells, inducing a 1.5- to 2-fold decrease in tumor burden, and increasing animal survival compared to carriers without drugs. A single-chain polypeptide containing the LDLR ligand Seq-1 was designed to form protein-only nanoparticles to successfully cross the BBB [177]. The ad of Seq-1 ligand increased the cell penetrability compared to the monomeric version of the nanoparticles in vitro. However, the enhancement was not observed after systemic administration. Beyond the authors’ suggestions for future studies, it should be of great interest to apply circadian rhythm studies to this nanoformulation, as the circadian expression of LDLR could be interfering in the particle’s uptake. Altogether, these are examples of nanoformulations that can be considered to have circadian rhythm internalization and whose therapeutic effect can be improved after taking this aspect into account. 

## 7. Conclusions and Future Perspectives

Developing therapeutic strategies for neurological disorders is still a challenging research area. In this field, scientists are trying to develop new therapeutic strategies to circumvent brain barriers, and are finding alternative routes through which to deliver therapeutic agents to the brain. In this review, we focused our attention on nanoformulations and their conjugation with a chronobiology approach to overcome the protection conferred by the brain barriers. 

Circadian rhythms in membrane transporters capable of transporting a great variety of drugs have been identified in brain barriers [11,12]. Therefore, the first notion that can be drawn is that the characterization of the influence of molecular clocks on the physiological functions of the barriers opens the possibility to test whether time matters when it comes to circumventing brain barriers. The second opinion that we can take from the gathered data is that, in general terms, nanoformulations are able to enhance the brain delivery of therapeutic agents. However, we also note that the connection between chronobiology and nanocarriers for drug delivery to the brain is an unexplored field. For example, in cancer cell lines, the uptake of methotrexate and p53 protein levels shows daily differences after the transfection with a polyethylenimine/pDNA (containing the p53 gene)/methotrexate nanoformulation at different time points [187]. This makes the conjugation between nanoformulations and chronobiology a potential strategy to be researched in order to improve the therapies for neurological disorders. In this review, we also report some nanoformulations with the potential to be tested with a chronobiological approach, and, in this phase, in vitro studies need to be performed. 

At the systemic level, the question addressed in this manuscript is more complex. The first point is that the efficacy of a drug depends on the pharmacokinetic and pharmacodynamic processes. As an example, a drug response could be influenced by a variety of factors, like the levels of metabolic enzymes, pH, and renal blood flow [188]. If various factors undergo circadian variations, the best time for drug administration would be a result of the rhythms of each factor. Thus, the rhythms of the various pharmacokinetic and pharmacodynamic factors should also be accessed. Here, the use of nanoformulations could also have an advantageous point. Besides the benefit of modulating drug distribution, nanoformulations may possibly be used to regulate the absorption, metabolism, and elimination processes in order to turn the daily variations in these processes into daily constants. Furthermore, the second point which can bring complexity to this question involves disease states, which have the ability to impact circadian rhythms, making it necessary to implement the circadian profile according to each disease context. 

In summary, as our final considerations, we conclude that the conjugation of nanoformulations with chronobiology has potential for the development and improvement of therapeutic strategies for neurological disorders. However, this research field is practically unexplored, and a significant amount of research still needs to be conducted.

## Figures and Tables

**Table 1:** Summary of the studies involving circadian rhythms in the blood–brain barrier (BBB).

Topic	Species/Cell Line	Results	References
Clock genes	C57BL/6J mice background (Bmal1^fl/fl^ (control) and Bmal1^fl/fl^; Tie2: Cre)	The following genes were rhythmically expressed in mice brain endothelial cells: brain and muscle ARNT-like 1 (*Bmal1*), nuclear receptor subfamily 1 group D member 1 (*Nr1d1*), period (*Per*) 2, albumin D-box binding protein (*Dbp*), hepatic leukemia factor (*Hlf*), and thyrotroph embryonic factor (*Tef*). The rhythmicity of these genes was lost in mice with an endothelial-specific Bmal1 deletion.	[11]
	Male Wistar rats	Bmal was showed to be rhythmically expressed in the brain microvessels of Wistar rats.	[73]
	hCMED/D3 cell line	*Bmal1* gene transcript showed a circadian rhythm in the human brain microvascular endothelial cell line hCMED/D3.	[11]
Blood–brain barrier (BBB) permeability	C57BL/6J mice background (Bmal1^fl/fl^ (control) and Bmal1^fl/fl^; Nestin: Cre)	BBB permeability to Evans blue and biotin was increased in brain Bmal1 knock-out mice when compared to their littermate controls.	[71]
	C57BL/6J mice	BBB permeability to Evans blue was not significantly different from Bmal1 knock-out mice or their littermate controls.	[72]
ATP-binding cassette (ABC) transporters	Male Sprague–Dawley rats	Wistar rats showed higher brain permeability to the ABCB1 substrate MC225 at Zeitgeber time (ZT) 15.	[74]
	C57BL/6J mice background (Bmal1^fl/fl^ (control) and Bmal1^fl/fl^; VECadherin^ERT2^: CRE)	The deletion of Bmal1 in mice endothelial cells resulted in the abrogation of the circadian oscillations of ABCB1-mediated Rhodamine123 brain efflux.	[75]
	C57BL/6J mice background (Bmal1^fl/fl^ (control) and Bmal1^fl/fl^; Tie2: Cre)	The deletion of endothelial Bmal1 resulted in the loss of the rhythmic oscillation in Rhodamine B (substrate of ABCB1) brain efflux. However, the expression of the two rodent abcb1 genes (*abcb1a* and *abcb1b*) was not rhythmic either in the control mice or the mice with a specific deletion for endothelial Bmal1.	[11]
	C57BL/6J mice background (Bmal1^fl/fl^ (control) and Bmal1^fl/fl^; Tie2: Cre)	Among various tested members of the ABC family, a microarray study reported that the *Abcg2* gene was the only one presenting circadian oscillations in brain endothelial cells. The circadian oscillation of *Abcg2* was not dependent on Bmal1.	[11]
	C57BL/6N male mice	A quantitative proteomic analysis showed that the protein expression of ABCB1 (a and b), ABCC4, ABCC9, and ABCG2 in mouse brain capillaries was not significantly different between ZT 0 and ZT 6, 12, and 18.	[76]
	hCMED/D3 cell line	*Abcb1* gene transcript and protein expression were not rhythmic in the human brain microvascular endothelial cell line hCMED/D3.	[11]
Solute carrier (SLC) transporters	C57BL/6J mice background (Bmal1^fl/fl^ (control) and Bmal1^fl/fl^; Tie2: Cre)	*Slc7a5*, which encodes for the large neutral amino acid transporter 1 small subunit 1, is rhythmically expressed in mouse brain endothelial cells. The rhythmic expression of *Slc7a5* is independent of Bmal1, since the expression was rhythmic either in brain endothelial cells from control mice or mice negative for endothelial Bmal1.	[11]
	C57BL/6N male mice	Among various SLC proteins tested, a quantitative proteomic analysis only showed differences for SLC9A3R2 expression between ZT0 and ZT6, 12, and 18.	[76]
Membrane receptors	C57BL/6N male mice	A quantitative proteomic analysis showed that the protein expression of insulin and transferrin receptors did not present any significant differences between ZT0 and ZT6, 12, and 18.	[76]
Tight junctions	C57BL/6N male mice	A proteomic analysis showed no significant differences in protein expression for claudin 5, occludin, and tight junction protein 1 between ZT0 and ZT6, 12, and 18.	[76]

**Table 3:** Examples of nanoformulations applied in CNS diseases for which barrier transportation mediators may possibly have expressions that follow a circadian rhythm.

Nanoformulation	Circadian-Expressed Receptor	Results	References
Docetaxel (DTX)-loaded solid lipidic nanoparticle surface modified with mannose	GLUT1	- Cytotoxicity and cell uptake unveiled enhanced efficacy - Increased DTX drug concentration in the brain	[178]
Ibuprofen-loaded brain-targeting magnetic nanoparticles (AA-Ibu-PEG-DA@MNPs) modified with ascorbic acid (AA)		- Limited cytotoxicity against brain endothelial cells - Nanoparticles were able to release ibuprofen into mouse plasma and brain homogenate	[179]
SiNPs-based nanoprobes bearing the ligand of glucosamine (G) and indocyanine green (ICG)		- Accumulation in glioblastoma tissues in mice - Superior photothermal effects of G-ICG-SiNPs in vivo	[180]
Betreliesoxybutyric acid (HBA) grafted docetaxel-loaded solid lipid nanoparticles (HD-SLNs)	MCT1	- Controlled release of DTX in vitro - Increased uptake of HD-SLNs from brain endothelial cells compared with Taxotere^®^	[181]
Cell-penetrating peptide (WRAP5) bearing Tf ligand and loading p53 plasmid	TfR	- Efficient glioblastoma cell penetration and consequent plasmid delivery into the nucleus - Decrease of tumor cells’ viability in vitro	[182]
Clofazimine-loaded PLGA-PEG nanoparticles (NP-CFZ) functionalized with the Tf ligand		- Superior brain endothelial hCMEC/D3 cells interaction and higher CFZ permeability across cell monolayers compared to the non-functionalized nanoparticles	[183]
Polylactic acid (PLA)-coated mesoporous silica nanoparticles (MSNPs) conjugated with a ligand peptide of LDLR for resveratrol delivery	LDLR	- Nanoparticles were able to cross the endothelial cell monolayer in an in vitro BBB model - Resveratrol was released and efficiently reduced the activation of microglia cells	[184]
LDLR ligand-functionalized gold nanoparticles (ApoB@AuNPs)		- ApoB@AuNPs were selectively captured by endothelial cells and pericyte and bulk tumor volume	[185]
PLGA nanoparticles functionalized with an apolipoprotein E-modified peptide (pep-apoE) or with lipocalin-type prostaglandin-d-synthase (L-PGDS)		- Non-functionalized and functionalized NPs did not injure dendritic cells’ viability and did not induced a proinflammatory profile from them - Functionalized NPs reached cerebral cortex parenchyma in mice after 2 h and were more significantly internalized by neurons and microglia	[186]

## References

[1] Dou X., Meints G.A., Sedaghat-Herati R. (2019). New Insights into the Interactions of a DNA Oligonucleotide with mPEGylated-PAMAM by Circular Dichroism and Solution NMR. J. Phys. Chem. B.

[2] Howes O.D., Mehta M.A. (2021). Challenges in CNS drug development and the role of imaging. Psychopharmacology.

[3] Daneman R., Prat A. (2015). The blood-brain barrier. Cold Spring Harb. Perspect. Biol..

[4] Santos C.R., Duarte A.C., Quintela T., Tomás J., Albuquerque T., Marques F., Palha J.A., Gonçalves I. (2017). The choroid plexus as a sex hormone target: Functional implications. Front. Neuroendocrinol..

[5] Bhadra U., Thakkar N., Das P., Pal Bhadra M. (2017). Evolution of circadian rhythms: From bacteria to human. Sleep Med..

[6] Panda S., Hogenesch J.B., Kay S.A. (2002). Circadian rhythms from flies to human. Nature.

[7] Takahashi J.S. (2017). Transcriptional architecture of the mammalian circadian clock. Nat. Rev. Genet..

[8] Hastings M.H., Maywood E.S., Brancaccio M. (2018). Generation of circadian rhythms in the suprachiasmatic nucleus. Nat. Rev. Neurosci..

[9] Quintela T., Albuquerque T., Lundkvist G., Carmine Belin A., Talhada D., Gonçalves I., Carro E., Santos C.R.A. (2018). The choroid plexus harbors a circadian oscillator modulated by estrogens. Chronobiol. Int..

[10] Quintela T., Sousa C., Patriarca F.M., Gonçalves I., Santos C.R. (2015). Gender associated circadian oscillations of the clock genes in rat choroid plexus. Brain Struct. Funct..

[11] Zhang S.L., Lahens N.F., Yue Z., Arnold D.M., Pakstis P.P., Schwarz J.E., Sehgal A. (2021). A circadian clock regulates efflux by the blood-brain barrier in mice and human cells. Nat. Commun..

[12] Furtado A., Mineiro R., Duarte A.C., Gonçalves I., Santos C.R., Quintela T. (2022). The Daily Expression of ABCC4 at the BCSFB Affects the Transport of Its Substrate Methotrexate. Int. J. Mol. Sci..

[13] Mudshinge S.R., Deore A.B., Patil S., Bhalgat C.M. (2011). Nanoparticles: Emerging carriers for drug delivery. Saudi Pharm. J..

[14] Pulgar V.M. (2018). Transcytosis to Cross the Blood Brain Barrier, New Advancements and Challenges. Front. Neurosci..

[15] Fang Z., Chen S., Qin J., Chen B., Ni G., Chen Z., Zhou J., Li Z., Ning Y., Wu C. (2016). Pluronic P85-coated poly(butylcyanoacrylate) nanoparticles overcome phenytoin resistance in P-glycoprotein overexpressing rats with lithium-pilocarpine-induced chronic temporal lobe epilepsy. Biomaterials.

[16] Kadry H., Noorani B., Cucullo L. (2020). A blood-brain barrier overview on structure, function, impairment, and biomarkers of integrity. Fluids Barriers CNS.

[17] Obermeier B., Verma A., Ransohoff R.M. (2016). The blood-brain barrier. Handb. Clin. Neurol..

[18] Serlin Y., Shelef I., Knyazer B., Friedman A. (2015). Anatomy and physiology of the blood-brain barrier. Semin. Cell Dev. Biol..

[19] Abbott N.J., Rönnbäck L., Hansson E. (2006). Astrocyte-endothelial interactions at the blood-brain barrier. Nat. Rev. Neurosci..

[20] Armulik A., Genové G., Mäe M., Nisancioglu M.H., Wallgard E., Niaudet C., He L., Norlin J., Lindblom P., Strittmatter K. (2010). Pericytes regulate the blood-brain barrier. Nature.

[21] Brown L.S., Foster C.G., Courtney J.M., King N.E., Howells D.W., Sutherland B.A. (2019). Pericytes and Neurovascular Function in the Healthy and Diseased Brain. Front. Cell. Neurosci..

[22] Krause G., Winkler L., Mueller S.L., Haseloff R.F., Piontek J., Blasig I.E. (2008). Structure and function of claudins. Biochim. Biophys. Acta.

[23] Vermette D., Hu P., Canarie M.F., Funaro M., Glover J., Pierce R.W. (2018). Tight junction structure, function, and assessment in the critically ill: A systematic review. Intensive Care Med. Exp..

[24] Günzel D., Yu A.S. (2013). Claudins and the modulation of tight junction permeability. Physiol. Rev..

[25] Berndt P., Winkler L., Cording J., Breitkreuz-Korff O., Rex A., Dithmer S., Rausch V., Blasig R., Richter M., Sporbert A. (2019). Tight junction proteins at the blood-brain barrier: Far more than claudin-5. Cell. Mol. Life Sci. CMLS.

[26] Greene C., Hanley N., Campbell M. (2019). Claudin-5: Gatekeeper of neurological function. Fluids Barriers CNS.

[27] Nitta T., Hata M., Gotoh S., Seo Y., Sasaki H., Hashimoto N., Furuse M., Tsukita S. (2003). Size-selective loosening of the blood-brain barrier in claudin-5-deficient mice. J. Cell Biol..

[28] Cooray H.C., Blackmore C.G., Maskell L., Barrand M.A. (2002). Localisation of breast cancer resistance protein in microvessel endothelium of human brain. Neuroreport.

[29] Daood M., Tsai C., Ahdab-Barmada M., Watchko J.F. (2008). ABC transporter (P-gp/ABCB1, MRP1/ABCC1, BCRP/ABCG2) expression in the developing human CNS. Neuropediatrics.

[30] Nies A.T., Jedlitschky G., König J., Herold-Mende C., Steiner H.H., Schmitt H.P., Keppler D. (2004). Expression and immunolocalization of the multidrug resistance proteins, MRP1-MRP6 (ABCC1-ABCC6), in human brain. Neuroscience.

[31] Virgintino D., Robertson D., Errede M., Benagiano V., Girolamo F., Maiorano E., Roncali L., Bertossi M. (2002). Expression of P-glycoprotein in human cerebral cortex microvessels. J. Histochem. Cytochem..

[32] Dauchy S., Dutheil F., Weaver R.J., Chassoux F., Daumas-Duport C., Couraud P.O., Scherrmann J.M., De Waziers I., Declèves X. (2008). ABC transporters, cytochromes P450 and their main transcription factors: Expression at the human blood-brain barrier. J. Neurochem..

[33] Spoletini I., Vitale C., Malorni W., Rosano G.M. (2012). Sex differences in drug effects: Interaction with sex hormones in adult life. Handb. Exp. Pharmacol..

[34] Sato R., Ohmori K., Umetsu M., Takao M., Tano M., Grant G., Porter B., Bet A., Terasaki T., Uchida Y. (2021). An Atlas of the Quantitative Protein Expression of Anti-Epileptic-Drug Transporters, Metabolizing Enzymes and Tight Junctions at the Blood-Brain Barrier in Epileptic Patients. Pharmaceutics.

[35] Zhao Z., Nelson A.R., Betsholtz C., Zlokovic B.V. (2015). Establishment and Dysfunction of the Blood-Brain Barrier. Cell.

[36] Chow B.W., Gu C. (2015). The molecular constituents of the blood-brain barrier. Trends Neurosci..

[37] Ghersi-Egea J.F., Strazielle N., Catala M., Silva-Vargas V., Doetsch F., Engelhardt B. (2018). Molecular anatomy and functions of the choroidal blood-cerebrospinal fluid barrier in health and disease. Acta Neuropathol..

[38] Liddelow S.A. (2015). Development of the choroid plexus and blood-CSF barrier. Front. Neurosci..

[39] Wolburg H., Paulus W. (2010). Choroid plexus: Biology and pathology. Acta Neuropathol..

[40] Praetorius J., Damkier H.H. (2017). Transport across the choroid plexus epithelium. Am. J. Physiol. Cell Physiol..

[41] Wichmann T.O., Damkier H.H., Pedersen M. (2021). A Brief Overview of the Cerebrospinal Fluid System and Its Implications for Brain and Spinal Cord Diseases. Front. Hum. Neurosci..

[42] Rosenthal R., Milatz S., Krug S.M., Oelrich B., Schulzke J.D., Amasheh S., Günzel D., Fromm M. (2010). Claudin-2, a component of the tight junction, forms a paracellular water channel. J. Cell Sci..

[43] Bernd A., Ott M., Ishikawa H., Schroten H., Schwerk C., Fricker G. (2015). Characterization of efflux transport proteins of the human choroid plexus papilloma cell line HIBCPP, a functional in vitro model of the blood-cerebrospinal fluid barrier. Pharm. Res..

[44] Leggas M., Adachi M., Scheffer G.L., Sun D., Wielinga P., Du G., Mercer K.E., Zhuang Y., Panetta J.C., Johnston B. (2004). Mrp4 confers resistance to topotecan and protects the brain from chemotherapy. Mol. Cell. Biol..

[45] Rao V.V., Dahlheimer J.L., Bardgett M.E., Snyder A.Z., Finch R.A., Sartorelli A.C., Piwnica-Worms D. (1999). Choroid plexus epithelial expression of MDR1 P glycoprotein and multidrug resistance-associated protein contribute to the blood-cerebrospinal-fluid drug-permeability barrier. Proc. Natl. Acad. Sci. USA.

[46] Tachikawa M., Watanabe M., Hori S., Fukaya M., Ohtsuki S., Asashima T., Terasaki T. (2005). Distinct spatio-temporal expression of ABCA and ABCG transporters in the developing and adult mouse brain. J. Neurochem..

[47] Ho H.T., Dahlin A., Wang J. (2012). Expression Profiling of Solute Carrier Gene Families at the Blood-CSF Barrier. Front. Pharmacol..

[48] Castañeyra-Ruiz L., González-Marrero I., Hernández-Abad L.G., Carmona-Calero E.M., Meyer G., Castañeyra-Perdomo A. (2016). A Distal to Proximal Gradient of Human Choroid Plexus Development, with Antagonistic Expression of Glut1 and AQP1 in Mature Cells vs. Calbindin and PCNA in Proliferative Cells. Front. Neuroanat..

[49] Damkier H.H., Nielsen S., Praetorius J. (2007). Molecular expression of SLC4-derived Na^+^-dependent anion transporters in selected human tissues. Am. J. Physiol. Regul. Integr. Comp. Physiol..

[50] Hinken M., Halwachs S., Kneuer C., Honscha W. (2011). Subcellular localization and distribution of the reduced folate carrier in normal rat tissues. Eur. J. Histochem. EJH.

[51] Roberts L.M., Black D.S., Raman C., Woodford K., Zhou M., Haggerty J.E., Yan A.T., Cwirla S.E., Grindstaff K.K. (2008). Subcellular localization of transporters along the rat blood-brain barrier and blood-cerebral-spinal fluid barrier by in vivo biotinylation. Neuroscience.

[52] Roberts L.M., Woodford K., Zhou M., Black D.S., Haggerty J.E., Tate E.H., Grindstaff K.K., Mengesha W., Raman C., Zerangue N. (2008). Expression of the thyroid hormone transporters monocarboxylate transporter-8 (SLC16A2) and organic ion transporter-14 (SLCO1C1) at the blood-brain barrier. Endocrinology.

[53] Strazielle N., Ghersi-Egea J.F. (2016). Potential Pathways for CNS Drug Delivery Across the Blood-Cerebrospinal Fluid Barrier. Curr. Pharm. Des..

[54] Mikitsh J.L., Chacko A.M. (2014). Pathways for small molecule delivery to the central nervous system across the blood-brain barrier. Perspect. Med. Chem..

[55] ter Beek J., Guskov A., Slotboom D.J. (2014). Structural diversity of ABC transporters. J. Gen. Physiol..

[56] Gil-Martins E., Barbosa D.J., Silva V., Remião F., Silva R. (2020). Dysfunction of ABC transporters at the blood-brain barrier: Role in neurological disorders. Pharm. Ther..

[57] Lin L., Yee S.W., Kim R.B., Giacomini K.M. (2015). SLC transporters as therapeutic targets: Emerging opportunities. Nat. Rev. Drug Discov..

[58] Colas C., Ung P.M., Schlessinger A. (2016). SLC Transporters: Structure, Function, and Drug Discovery. Medchemcomm.

[59] Dickens D., Webb S.D., Antonyuk S., Giannoudis A., Owen A., Rädisch S., Hasnain S.S., Pirmohamed M. (2013). Transport of gabapentin by LAT1 (SLC7A5). Biochem. Pharmacol..

[60] Kageyama T., Nakamura M., Matsuo A., Yamasaki Y., Takakura Y., Hashida M., Kanai Y., Naito M., Tsuruo T., Minato N. (2000). The 4F2hc/LAT1 complex transports L-DOPA across the blood-brain barrier. Brain Res..

[61] Duelli R., Enerson B.E., Gerhart D.Z., Drewes L.R. (2000). Expression of large amino acid transporter LAT1 in rat brain endothelium. J. Cereb. Blood Flow Metab..

[62] Singh N., Ecker G.F. (2018). Insights into the Structure, Function, and Ligand Discovery of the Large Neutral Amino Acid Transporter 1, LAT1. Int. J. Mol. Sci..

[63] Morris M.E., Rodriguez-Cruz V., Felmlee M.A. (2017). SLC and ABC Transporters: Expression, Localization, and Species Differences at the Blood-Brain and the Blood-Cerebrospinal Fluid Barriers. AAPS J..

[64] Stieger B., Gao B. (2015). Drug transporters in the central nervous system. Clin. Pharmacokinet..

[65] Puris E., Fricker G., Gynther M. (2022). Targeting Transporters for Drug Delivery to the Brain: Can We Do Better?. Pharm. Res..

[66] Azarmi M., Maleki H., Nikkam N., Malekinejad H. (2020). Transcellular brain drug delivery: A review on recent advancements. Int. J. Pharm..

[67] Grapp M., Wrede A., Schweizer M., Hüwel S., Galla H.J., Snaidero N., Simons M., Bückers J., Low P.S., Urlaub H. (2013). Choroid plexus transcytosis and exosome shuttling deliver folate into brain parenchyma. Nat. Commun..

[68] Hervé F., Ghinea N., Scherrmann J.M. (2008). CNS delivery via adsorptive transcytosis. AAPS J..

[69] Vorbrodt A.W. (1989). Ultracytochemical characterization of anionic sites in the wall of brain capillaries. J. Neurocytol..

[70] Zhu X., Jin K., Huang Y., Pang Z., Gao H., Gao X. (2019). 7-Brain drug delivery by adsorption-mediated transcytosis. Brain Targeted Drug Delivery System.

[71] Nakazato R., Kawabe K., Yamada D., Ikeno S., Mieda M., Shimba S., Hinoi E., Yoneda Y., Takarada T. (2017). Disruption of Bmal1 Impairs Blood-Brain Barrier Integrity via Pericyte Dysfunction. J. Neurosci..

[72] Hemmeryckx B., Frederix L., Lijnen H.R. (2019). Deficiency of Bmal1 disrupts the diurnal rhythm of haemostasis. Exp. Gerontol..

[73] Szczepkowska A., Harazin A., Barna L., Deli M.A., Skipor J. (2021). Identification of Reference Genes for Circadian Studies on Brain Microvessels and Choroid Plexus Samples Isolated from Rats. Biomolecules.

[74] Savolainen H., Meerlo P., Elsinga P.H., Windhorst A.D., Dierckx R.A., Colabufo N.A., van Waarde A., Luurtsema G. (2016). P-glycoprotein Function in the Rodent Brain Displays a Daily Rhythm, a Quantitative In Vivo PET Study. AAPS J..

[75] Pulido R.S., Munji R.N., Chan T.C., Quirk C.R., Weiner G.A., Weger B.D., Rossi M.J., Elmsaouri S., Malfavon M., Deng A. (2020). Neuronal Activity Regulates Blood-Brain Barrier Efflux Transport through Endothelial Circadian Genes. Neuron.

[76] Ogata S., Ito S., Masuda T., Ohtsuki S. (2022). Diurnal Changes in Protein Expression at the Blood-Brain Barrier in Mice. Biol. Pharm. Bull..

[77] Zhang S.L., Yue Z., Arnold D.M., Artiushin G., Sehgal A. (2018). A Circadian Clock in the Blood-Brain Barrier Regulates Xenobiotic Efflux. Cell.

[78] Voog L., Eriksson T. (1992). Relationship between plasma and brain large neutral amino acids in rats fed diets with different compositions at different times of the day. J. Neurochem..

[79] Jiang X., Liu Y., Zhang X.Y., Liu X., Liu X., Wu X., Jose P.A., Duan S., Xu F.J., Yang Z. (2022). Intestinal Gastrin/CCKBR (Cholecystokinin B Receptor) Ameliorates Salt-Sensitive Hypertension by Inhibiting Intestinal Na(+)/H(+) Exchanger 3 Activity Through a PKC (Protein Kinase C)-Mediated NHERF1 and NHERF2 Pathway. Hypertension.

[80] Gómez-González B., Hurtado-Alvarado G., Esqueda-León E., Santana-Miranda R., Rojas-Zamorano J., Velázquez-Moctezuma J. (2013). REM sleep loss and recovery regulates blood-brain barrier function. Curr. Neurovasc. Res..

[81] Banks W.A., Kastin A.J., Ehrensing C.A. (1998). Diurnal uptake of circulating interleukin-1alpha by brain, spinal cord, testis and muscle. Neuroimmunomodulation.

[82] Skinner R.A., Gibson R.M., Rothwell N.J., Pinteaux E., Penny J.I. (2009). Transport of interleukin-1 across cerebromicrovascular endothelial cells. Br. J. Pharmacol..

[83] Kress G.J., Liao F., Dimitry J., Cedeno M.R., FitzGerald G.A., Holtzman D.M., Musiek E.S. (2018). Regulation of amyloid-β dynamics and pathology by the circadian clock. J. Exp. Med..

[84] Liška K., Sládek M., Čečmanová V., Sumová A. (2021). Glucocorticoids reset circadian clock in choroid plexus via period genes. J. Endocrinol..

[85] Yamaguchi T., Hamada T., Matsuzaki T., Iijima N. (2020). Characterization of the circadian oscillator in the choroid plexus of rats. Biochem. Biophys. Res. Commun..

[86] Quintela T., Marcelino H., Deery M.J., Feret R., Howard J., Lilley K.S., Albuquerque T., Gonçalves I., Duarte A.C., Santos C.R. (2016). Sex-Related Differences in Rat Choroid Plexus and Cerebrospinal Fluid: A cDNA Microarray and Proteomic Analysis. J. Neuroendocrinol..

[87] Quintela T., Gonçalves I., Carreto L.C., Santos M.A., Marcelino H., Patriarca F.M., Santos C.R. (2013). Analysis of the effects of sex hormone background on the rat choroid plexus transcriptome by cDNA microarrays. PLoS ONE.

[88] Myung J., Schmal C., Hong S., Tsukizawa Y., Rose P., Zhang Y., Holtzman M.J., De Schutter E., Herzel H., Bordyugov G. (2018). The choroid plexus is an important circadian clock component. Nat. Commun..

[89] Yamaguchi T., Hamada T., Iijima N. (2022). Differences in recovery processes of circadian oscillators in various tissues after sevoflurane treatment in vivo. Biochem. Biophys. Rep..

[90] Quintela T., Furtado A., Duarte A.C., Gonçalves I., Myung J., Santos C.R.A. (2021). The role of circadian rhythm in choroid plexus functions. Prog. Neurobiol..

[91] Furtado A., Esgalhado A.J., Duarte A.C., Costa A.R., Costa-Brito A.R., Carro E., Ishikawa H., Schroten H., Schwerk C., Gonçalves I. (2023). Circadian rhythmicity of amyloid-beta-related molecules is disrupted in the choroid plexus of a female Alzheimer’s disease mouse model. J. Neurosci. Res..

[92] Oda T., Pasinetti G.M., Osterburg H.H., Anderson C., Johnson S.A., Finch C.E. (1994). Purification and characterization of brain clusterin. Biochem. Biophys. Res. Commun..

[93] Oda T., Wals P., Osterburg H.H., Johnson S.A., Pasinetti G.M., Morgan T.E., Rozovsky I., Stine W.B., Snyder S.W., Holzman T.F. (1995). Clusterin (apoJ) alters the aggregation of amyloid beta-peptide (A beta 1–42) and forms slowly sedimenting A beta complexes that cause oxidative stress. Exp. Neurol..

[94] Duarte A.C., Furtado A., Hrynchak M.V., Costa A.R., Talhada D., Gonçalves I., Lemos M.C., Quintela T., Santos C.R.A. (2020). Age, Sex Hormones, and Circadian Rhythm Regulate the Expression of Amyloid-Beta Scavengers at the Choroid Plexus. Int. J. Mol. Sci..

[95] Costa R., Gonçalves A., Saraiva M.J., Cardoso I. (2008). Transthyretin binding to A-Beta peptide--impact on A-Beta fibrillogenesis and toxicity. FEBS Lett..

[96] Li X., Zhang X., Ladiwala A.R., Du D., Yadav J.K., Tessier P.M., Wright P.E., Kelly J.W., Buxbaum J.N. (2013). Mechanisms of transthyretin inhibition of β-amyloid aggregation in vitro. J. Neurosci..

[97] Xie L., Kang H., Xu Q., Chen M.J., Liao Y., Thiyagarajan M., O’Donnell J., Christensen D.J., Nicholson C., Iliff J.J. (2013). Sleep drives metabolite clearance from the adult brain. Science.

[98] Furtado A., Astaburuaga R., Costa A., Duarte A.C., Gonçalves I., Cipolla-Neto J., Lemos M.C., Carro E., Relógio A., Santos C.R.A. (2020). The Rhythmicity of Clock Genes is Disrupted in the Choroid Plexus of the APP/PS1 Mouse Model of Alzheimer’s Disease. J. Alzheimer’s Dis..

[99] Quintela T., Gonçalves I., Silva M., Duarte A.C., Guedes P., Andrade K., Freitas F., Talhada D., Albuquerque T., Tavares S. (2018). Choroid plexus is an additional source of melatonin in the brain. J. Pineal Res..

[100] Lagaraine C., Skipor J., Szczepkowska A., Dufourny L., Thiery J.C. (2011). Tight junction proteins vary in the choroid plexus of ewes according to photoperiod. Brain Res..

[101] Domínguez A., Suárez-Merino B., Goñi-de-Cerio F. (2014). Nanoparticles and blood-brain barrier: The key to central nervous system diseases. J. Nanosci. Nanotechnol..

[102] Borsook D. (2012). Neurological diseases and pain. Brain.

[103] Urquhart B.L., Kim R.B. (2009). Blood-brain barrier transporters and response to CNS-active drugs. Eur. J. Clin. Pharmacol..

[104] Gribkoff V.K., Kaczmarek L.K. (2017). The need for new approaches in CNS drug discovery: Why drugs have failed, and what can be done to improve outcomes. Neuropharmacology.

[105] Jeevanandam J., Barhoum A., Chan Y.S., Dufresne A., Danquah M.K. (2018). Review on nanoparticles and nanostructured materials: History, sources, toxicity and regulations. Beilstein J. Nanotechnol..

[106] Shen Z., Nieh M.P., Li Y. (2016). Decorating Nanoparticle Surface for Targeted Drug Delivery: Opportunities and Challenges. Polymers.

[107] Zhang R.X., Wong H.L., Xue H.Y., Eoh J.Y., Wu X.Y. (2016). Nanomedicine of synergistic drug combinations for cancer therapy-Strategies and perspectives. J. Control. Release.

[108] Ayub A., Wettig S. (2022). An Overview of Nanotechnologies for Drug Delivery to the Brain. Pharmaceutics.

[109] Zhang W., Mehta A., Tong Z., Esser L., Voelcker N.H. (2021). Development of Polymeric Nanoparticles for Blood–Brain Barrier Transfer—Strategies and Challenges. Adv. Sci..

[110] Juhairiyah F., de Lange E.C.M. (2021). Understanding Drug Delivery to the Brain Using Liposome-Based Strategies: Studies that Provide Mechanistic Insights Are Essential. AAPS J..

[111] Azam N., Najabat Ali M., Javaid Khan T. (2021). Carbon Quantum Dots for Biomedical Applications: Review and Analysis. Front. Mater..

[112] Ferreira Soares D.C., Domingues S.C., Viana D.B., Tebaldi M.L. (2020). Polymer-hybrid nanoparticles: Current advances in biomedical applications. Biomed. Pharmacother..

[113] Zielińska A., Carreiró F., Oliveira A.M., Neves A., Pires B., Venkatesh D.N., Durazzo A., Lucarini M., Eder P., Silva A.M. (2020). Polymeric Nanoparticles: Production, Characterization, Toxicology and Ecotoxicology. Molecules.

[114] La Barbera L., Mauri E., D’Amelio M., Gori M. (2022). Functionalization strategies of polymeric nanoparticles for drug delivery in Alzheimer’s disease: Current trends and future perspectives. Front. Neurosci..

[115] Caraway C.A., Gaitsch H., Wicks E.E., Kalluri A., Kunadi N., Tyler B.M. (2022). Polymeric Nanoparticles in Brain Cancer Therapy: A Review of Current Approaches. Polymers.

[116] Sartaj A., Qamar Z., Md S., Alhakamy N.A., Baboota S., Ali J. (2022). An Insight to Brain Targeting Utilizing Polymeric Nanoparticles: Effective Treatment Modalities for Neurological Disorders and Brain Tumor. Front. Bioeng. Biotechnol..

[117] Islam Y., Leach A.G., Smith J., Pluchino S., Coxonl C.R., Sivakumaran M., Downing J., Fatokun A.A., Teixidò M., Ehtezazi T. (2020). Peptide based drug delivery systems to the brain. Nano Express.

[118] Hartl N., Adams F., Merkel O.M. (2021). From Adsorption to Covalent Bonding: Apolipoprotein E Functionalization of Polymeric Nanoparticles for Drug Delivery Across the Blood–Brain Barrier. Adv. Ther..

[119] Nabi B., Rehman S., Fazil M., Khan S., Baboota S., Ali J. (2020). Riluzole-loaded nanoparticles to alleviate the symptoms of neurological disorders by attenuating oxidative stress. Drug Dev. Ind. Pharm..

[120] Neves A.R., Queiroz J.F., Lima S.A.C., Reis S. (2017). Apo E-Functionalization of Solid Lipid Nanoparticles Enhances Brain Drug Delivery: Uptake Mechanism and Transport Pathways. Bioconjug. Chem..

[121] Suk J.S., Xu Q., Kim N., Hanes J., Ensign L.M. (2016). PEGylation as a strategy for improving nanoparticle-based drug and gene delivery. Adv. Drug Deliv. Rev..

[122] Sun D., Xue A., Zhang B., Lou H., Shi H., Zhang X. (2015). Polysorbate 80-coated PLGA nanoparticles improve the permeability of acetylpuerarin and enhance its brain-protective effects in rats. J. Pharm. Pharmacol..

[123] Di Mauro P.P., Cascante A., Brugada Vilà P., Gómez-Vallejo V., Llop J., Borrós S. (2018). Peptide-functionalized and high drug loaded novel nanoparticles as dual-targeting drug delivery system for modulated and controlled release of paclitaxel to brain glioma. Int. J. Pharm..

[124] Gothwal A., Kumar H., Nakhate K.T., Ajazuddin, Dutta A., Borah A., Gupta U. (2019). Lactoferrin Coupled Lower Generation PAMAM Dendrimers for Brain Targeted Delivery of Memantine in Aluminum-Chloride-Induced Alzheimer’s Disease in Mice. Bioconjug. Chem..

[125] Khan S.A., Shah M.R., Imran M., Ullah S. (2020). Chapter 1-Metal nanoparticles toxicity: Role of physicochemical aspects. Metal Nanoparticles for Drug Delivery and Diagnostic Applications.

[126] Piñón-Segundo E., Mendoza-Muñoz N., Quintanar-Guerrero D., Subramani K., Ahmed W., Hartsfield J.K. (2013). Chapter 23-Nanoparticles as Dental Drug-Delivery Systems. Nanobiomaterials in Clinical Dentistry.

[127] Chandrakala V., Aruna V., Angajala G. (2022). Review on metal nanoparticles as nanocarriers: Current challenges and perspectives in drug delivery systems. Emergent Mater..

[128] Yang Z., Liu Z.W., Allaker R.P., Reip P., Oxford J., Ahmad Z., Ren G. (2010). A review of nanoparticle functionality and toxicity on the central nervous system. J. R. Soc. Interface.

[129] Wang S., Zhang B., Su L., Nie W., Han D., Han G., Zhang H., Chong C., Tan J. (2019). Subcellular distributions of iron oxide nanoparticles in rat brains affected by different surface modifications. J. Biomed. Mater. Res. Part A.

[130] Hersh A.M., Alomari S., Tyler B.M. (2022). Crossing the Blood-Brain Barrier: Advances in Nanoparticle Technology for Drug Delivery in Neuro-Oncology. Int. J. Mol. Sci..

[131] Afzalipour R., Khoei S., Khoee S., Shirvalilou S., Raoufi N.J., Motevalian M., Karimi M.Y. (2021). Thermosensitive magnetic nanoparticles exposed to alternating magnetic field and heat-mediated chemotherapy for an effective dual therapy in rat glioma model. Nanomed. Nanotechnol. Biol. Med..

[132] Anselmo A.C., Mitragotri S. (2019). Nanoparticles in the clinic: An update. Bioeng. Transl. Med..

[133] Micheli M.R., Bova R., Magini A., Polidoro M., Emiliani C. (2012). Lipid-based nanocarriers for CNS-targeted drug delivery. Recent Pat. CNS Drug Discov..

[134] Amin M., Seynhaeve A.L.B., Sharifi M., Falahati M., ten Hagen T.L.M. (2022). Liposomal Drug Delivery Systems for Cancer Therapy: The Rotterdam Experience. Pharmaceutics.

[135] Liu P., Chen G., Zhang J. (2022). A Review of Liposomes as a Drug Delivery System: Current Status of Approved Products, Regulatory Environments, and Future Perspectives. Molecules.

[136] Schnyder A., Huwyler J. (2005). Drug transport to brain with targeted liposomes. NeuroRx.

[137] Johnsen K.B., Burkhart A., Melander F., Kempen P.J., Vejlebo J.B., Siupka P., Nielsen M.S., Andresen T.L., Moos T. (2017). Targeting transferrin receptors at the blood-brain barrier improves the uptake of immunoliposomes and subsequent cargo transport into the brain parenchyma. Sci. Rep..

[138] Johnsen K.B., Bak M., Melander F., Thomsen M.S., Burkhart A., Kempen P.J., Andresen T.L., Moos T. (2019). Modulating the antibody density changes the uptake and transport at the blood-brain barrier of both transferrin receptor-targeted gold nanoparticles and liposomal cargo. J. Control. Release.

[139] Zhang Z., Guan J., Jiang Z., Yang Y., Liu J., Hua W., Mao Y., Li C., Lu W., Qian J. (2019). Brain-targeted drug delivery by manipulating protein corona functions. Nat. Commun..

[140] Hu Y., Gaillard P.J., de Lange E.C.M., Hammarlund-Udenaes M. (2019). Targeted brain delivery of methotrexate by glutathione PEGylated liposomes: How can the formulation make a difference?. Eur. J. Pharm. Biopharm..

[141] Singh J., Nayak P., Singh G., Khandai M., Sarangi R.R., Kar M.K. (2023). Carbon Nanostructures as Therapeutic Cargoes: Recent Developments and Challenges. C.

[142] Dubey R., Dutta D., Sarkar A., Chattopadhyay P. (2021). Functionalized carbon nanotubes: Synthesis, properties and applications in water purification, drug delivery, and material and biomedical sciences. Nanoscale Adv..

[143] Zare H., Ahmadi S., Ghasemi A., Ghanbari M., Rabiee N., Bagherzadeh M., Karimi M., Webster T.J., Hamblin M.R., Mostafavi E. (2021). Carbon Nanotubes: Smart Drug/Gene Delivery Carriers. Int. J. Nanomed..

[144] Mohanta D., Patnaik S., Sood S., Das N. (2019). Carbon nanotubes:Evaluation of toxicity at biointerfaces. J. Pharm. Anal..

[145] Kafa H., Wang J.T., Rubio N., Venner K., Anderson G., Pach E., Ballesteros B., Preston J.E., Abbott N.J., Al-Jamal K.T. (2015). The interaction of carbon nanotubes with an in vitro blood-brain barrier model and mouse brain in vivo. Biomaterials.

[146] Kafa H., Wang J.T., Rubio N., Klippstein R., Costa P.M., Hassan H.A., Sosabowski J.K., Bansal S.S., Preston J.E., Abbott N.J. (2016). Translocation of LRP1 targeted carbon nanotubes of different diameters across the blood-brain barrier in vitro and in vivo. J. Control. Release.

[147] You Y., Wang N., He L., Shi C., Zhang D., Liu Y., Luo L., Chen T. (2019). Designing dual-functionalized carbon nanotubes with high blood–brain-barrier permeability for precise orthotopic glioma therapy. Dalton Trans..

[148] Harsha P.J., Thotakura N., Kumar M., Sharma S., Mittal A., Khurana R.K., Singh B., Negi P., Raza K. (2019). A novel PEGylated carbon nanotube conjugated mangiferin: An explorative nanomedicine for brain cancer cells. J. Drug Deliv. Sci. Technol..

[149] Guo Q., You H., Yang X., Lin B., Zhu Z., Lu Z., Li X., Zhao Y., Mao L., Shen S. (2017). Functional single-walled carbon nanotubes ‘CAR’ for targeting dopamine delivery into the brain of parkinsonian mice. Nanoscale.

[150] Qiao R., Jia Q., Hüwel S., Xia R., Liu T., Gao F., Galla H.-J., Gao M. (2012). Receptor-Mediated Delivery of Magnetic Nanoparticles across the Blood–Brain Barrier. ACS Nano.

[151] Wang J., Chen D., Ho E.A. (2021). Challenges in the development and establishment of exosome-based drug delivery systems. J. Control. Release.

[152] Yakubovich E.I., Polischouk A.G., Evtushenko V.I. (2022). Principles and Problems of Exosome Isolation from Biological Fluids. Biochem. Suppl. Ser. A Membr. Cell Biol..

[153] Heidarzadeh M., Gürsoy-Özdemir Y., Kaya M., Eslami Abriz A., Zarebkohan A., Rahbarghazi R., Sokullu E. (2021). Exosomal delivery of therapeutic modulators through the blood–brain barrier; promise and pitfalls. Cell Biosci..

[154] Matsumoto J., Stewart T., Banks W.A., Zhang J. (2017). The Transport Mechanism of Extracellular Vesicles at the Blood-Brain Barrier. Curr. Pharm. Des..

[155] Liu X., Xia T., Fang Y., Zuo H., Dong X., Xu P., Ouyang J. (2022). Overcoming the blood–brain barrier by using a multistage exosome delivery system to inhibit central nervous system lymphoma. Nanomed. Nanotechnol. Biol. Med..

[156] Karami Fath M., Azami J., Masoudi A., Mosaddeghi Heris R., Rahmani E., Alavi F., Alagheband Bahrami A., Payandeh Z., Khalesi B., Dadkhah M. (2022). Exosome-based strategies for diagnosis and therapy of glioma cancer. Cancer Cell Int..

[157] Jia G., Han Y., An Y., Ding Y., He C., Wang X., Tang Q. (2018). NRP-1 targeted and cargo-loaded exosomes facilitate simultaneous imaging and therapy of glioma in vitro and in vivo. Biomaterials.

[158] Peng H., Li Y., Ji W., Zhao R., Lu Z., Shen J., Wu Y., Wang J., Hao Q., Wang J. (2022). Intranasal Administration of Self-Oriented Nanocarriers Based on Therapeutic Exosomes for Synergistic Treatment of Parkinson’s Disease. ACS Nano.

[159] Patel A., Olang C.A., Lewis G., Mandalaneni K., Anand N., Gorantla V.R. (2022). An Overview of Parkinson’s Disease: Curcumin as a Possible Alternative Treatment. Cureus.

[160] Copeland C., Stabenfeldt S.E. (2020). Leveraging the Dynamic Blood-Brain Barrier for Central Nervous System Nanoparticle-based Drug Delivery Applications. Curr. Opin. Biomed. Eng..

[161] Kreuter J. (2015). Influence of chronobiology on the nanoparticle-mediated drug uptake into the brain. Pharmaceutics.

[162] Gonzalez-Carter D., Liu X., Tockary T.A., Dirisala A., Toh K., Anraku Y., Kataoka K. (2020). Targeting nanoparticles to the brain by exploiting the blood-brain barrier impermeability to selectively label the brain endothelium. Proc. Natl. Acad. Sci. USA.

[163] Kudo T., Kawashima M., Tamagawa T., Shibata S. (2008). Clock mutation facilitates accumulation of cholesterol in the liver of mice fed a cholesterol and/or cholic acid diet. Am. J. Physiol. Endocrinol. Metab..

[164] Lee Y.J., Han D.H., Pak Y.K., Cho S.H. (2012). Circadian regulation of low density lipoprotein receptor promoter activity by CLOCK/BMAL1, Hes1 and Hes6. Exp. Mol. Med..

[165] Okazaki F., Matsunaga N., Okazaki H., Azuma H., Hamamura K., Tsuruta A., Tsurudome Y., Ogino T., Hara Y., Suzuki T. (2016). Circadian Clock in a Mouse Colon Tumor Regulates Intracellular Iron Levels to Promote Tumor Progression. J. Biol. Chem..

[166] Rodríguez-Cortés B., Hurtado-Alvarado G., Martínez-Gómez R., León-Mercado L.A., Prager-Khoutorsky M., Buijs R.M. (2022). Suprachiasmatic nucleus-mediated glucose entry into the arcuate nucleus determines the daily rhythm in blood glycemia. Curr. Biol..

[167] Milićević N., Ten Brink J.B., Ten Asbroek A., Bergen A.A., Felder-Schmittbuhl M.P. (2020). The circadian clock regulates RPE-mediated lactate transport via SLC16A1 (MCT1). Exp. Eye Res..

[168] Soltésová D., Veselá A., Mravec B., Herichová I. (2013). Daily profile of glut1 and glut4 expression in tissues inside and outside the blood-brain barrier in control and streptozotocin-treated rats. Physiol. Res..

[169] Vagnerová K., Ergang P., Soták M., Balounová K., Kvapilová P., Vodička M., Pácha J. (2019). Diurnal expression of ABC and SLC transporters in jejunum is modulated by adrenalectomy. Comp. Biochem. Physiol. Toxicol. Pharmacol..

[170] Yuan P., Yang T., Mu J., Zhao J., Yang Y., Yan Z., Hou Y., Chen C., Xing J., Zhang H. (2020). Circadian clock gene NPAS2 promotes reprogramming of glucose metabolism in hepatocellular carcinoma cells. Cancer Lett..

[171] Min H.S., Kim H.J., Naito M., Ogura S., Toh K., Hayashi K., Kim B.S., Fukushima S., Anraku Y., Miyata K. (2020). Systemic Brain Delivery of Antisense Oligonucleotides across the Blood–Brain Barrier with a Glucose-Coated Polymeric Nanocarrier. Angew. Chem. Int. Ed..

[172] Anraku Y., Kuwahara H., Fukusato Y., Mizoguchi A., Ishii T., Nitta K., Matsumoto Y., Toh K., Miyata K., Uchida S. (2017). Glycaemic control boosts glucosylated nanocarrier crossing the BBB into the brain. Nat. Commun..

[173] Zhang L., Zhao Y., Yue Q., Fu Q., Hai L., Guo L., Wang Q., Wu Y. (2018). Preparation and Characterization of GLUT1-mediated Novel Brain Targeting Magnetic Nanoparticles. Lett. Drug Des. Discov..

[174] Ak G., Ünal A., Karakayalı T., Özel B., Selvi Günel N., Hamarat Şanlıer Ş. (2021). Brain-targeted, drug-loaded solid lipid nanoparticles against glioblastoma cells in culture. Colloids Surf. B Biointerfaces.

[175] Ruan S., Qin L., Xiao W., Hu C., Zhou Y., Wang R., Sun X., Yu W., He Q., Gao H. (2018). Acid-Responsive Transferrin Dissociation and GLUT Mediated Exocytosis for Increased Blood–Brain Barrier Transcytosis and Programmed Glioma Targeting Delivery. Adv. Funct. Mater..

[176] Lam F.C., Morton S.W., Wyckoff J., Vu Han T.-L., Hwang M.K., Maffa A., Balkanska-Sinclair E., Yaffe M.B., Floyd S.R., Hammond P.T. (2018). Enhanced efficacy of combined temozolomide and bromodomain inhibitor therapy for gliomas using targeted nanoparticles. Nat. Commun..

[177] Serna N., Céspedes M.V., Saccardo P., Xu Z., Unzueta U., Álamo P., Pesarrodona M., Sánchez-Chardi A., Roldán M., Mangues R. (2016). Rational engineering of single-chain polypeptides into protein-only, BBB-targeted nanoparticles. Nanomed. Nanotechnol. Biol. Med..

[178] Singh I., Swami R., Jeengar M.K., Khan W., Sistla R. (2015). p-Aminophenyl-α-d-mannopyranoside engineered lipidic nanoparticles for effective delivery of docetaxel to brain. Chem. Phys. Lipids.

[179] Wang L., Zhao Y., Lu R., Peng Y., Guo L., Hai L., Guan M., Wu Y. (2018). Preparation and Characterization of Novel Brain Targeting Magnetic Nanoparticles Modified with Ascorbic Acid. Nano.

[180] Sun R., Liu M., Xu Z., Song B., He Y., Wang H. (2022). Silicon-based nanoprobes cross the blood—Brain barrier for photothermal therapy of glioblastoma. Nano Res..

[181] Venishetty V.K., Samala R., Komuravelli R., Kuncha M., Sistla R., Diwan P.V. (2013). β-Hydroxybutyric acid grafted solid lipid nanoparticles: A novel strategy to improve drug delivery to brain. Nanomed. Nanotechnol. Biol. Med..

[182] Neves A.R., Albuquerque T., Faria R., Gonçalves A.M., Santos C., Vivès E., Boisguérin P., Passarinha L.A., Sousa Â., Costa D. (2022). Development of WRAP5 Peptide Complexes for Targeted Drug/Gene Co-Delivery toward Glioblastoma Therapy. Pharmaceutics.

[183] de Castro R.R., do Carmo F.A., Martins C., Simon A., de Sousa V.P., Rodrigues C.R., Cabral L.M., Sarmento B. (2021). Clofazimine functionalized polymeric nanoparticles for brain delivery in the tuberculosis treatment. Int. J. Pharm..

[184] Shen Y., Cao B., Snyder N.R., Woeppel K.M., Eles J.R., Cui X.T. (2018). ROS responsive resveratrol delivery from LDLR peptide conjugated PLA-coated mesoporous silica nanoparticles across the blood–brain barrier. J. Nanobiotechnol..

[185] Seo S., Kim E.H., Chang W.S., Lee W.S., Kim K.H., Kim J.K. (2022). Enhanced proton treatment with a LDLR-ligand peptide-conjugated gold nanoparticles targeting the tumor microenvironment in an infiltrative brain tumor model. Am. J. Cancer Res..

[186] Portioli C., Bovi M., Benati D., Donini M., Perduca M., Romeo A., Dusi S., Monaco H.L., Bentivoglio M. (2017). Novel functionalization strategies of polymeric nanoparticles as carriers for brain medications. J. Biomed. Mater. Res. Part A.

[187] Albuquerque T., Neves A.R., Quintela T., Costa D. (2022). The Influence of Circadian Rhythm on Cancer Cells Targeting and Transfection Efficiency of a Polycation-Drug/Gene Delivery Vector. Polymers.

[188] Gaspar L.S., Álvaro A.R., Carmo-Silva S., Mendes A.F., Relógio A., Cavadas C. (2019). The importance of determining circadian parameters in pharmacological studies. Br. J. Pharmacol..

